# NtrBC Selectively Regulates Host-Pathogen Interactions, Virulence, and Ciprofloxacin Susceptibility of *Pseudomonas aeruginosa*

**DOI:** 10.3389/fcimb.2021.694789

**Published:** 2021-06-24

**Authors:** Morgan A. Alford, Beverlie Baquir, Andy An, Ka-Yee G. Choi, Robert E. W. Hancock

**Affiliations:** Centre for Microbial Diseases and Immunity Research, University of British Columbia, Vancouver, BC, Canada

**Keywords:** nitrogen metabolism, virulence, abscess, respiratory infection, antibiotic resistance

## Abstract

*Pseudomonas aeruginosa* is a metabolically versatile opportunistic pathogen capable of infecting distinct niches of the human body, including skin wounds and the lungs of cystic fibrosis patients. Eradication of *P. aeruginosa* infection is becoming increasingly difficult due to the numerous resistance mechanisms it employs. Adaptive resistance is characterized by a transient state of decreased susceptibility to antibiotic therapy that is distinct from acquired or intrinsic resistance, can be triggered by various environmental stimuli and reverted by removal of the stimulus. Further, adaptive resistance is intrinsically linked to lifestyles such as swarming motility and biofilm formation, both of which are important in infections and lead to multi-drug adaptive resistance. Here, we demonstrated that NtrBC, the master of nitrogen control, had a selective role in host colonization and a substantial role in determining intrinsic resistance to ciprofloxacin. *P. aeruginosa* mutant strains (Δ*ntrB*, Δ*ntrC* and Δ*ntrBC*) colonized the skin but not the respiratory tract of mice as well as WT and, unlike WT, could be reduced or eradicated from the skin by ciprofloxacin. We hypothesized that nutrient availability contributed to these phenomena and found that susceptibility to ciprofloxacin was impacted by nitrogen source in laboratory media. *P. aeruginosa* Δ*ntrB*, Δ*ntrC* and Δ*ntrBC* also exhibited distinct host interactions, including modestly increased cytotoxicity toward human bronchial epithelial cells, reduced virulence factor production and 10-fold increased uptake by macrophages. These data might explain why NtrBC mutants were less adept at colonizing the upper respiratory tract of mice. Thus, NtrBC represents a link between nitrogen metabolism, adaptation and virulence of the pathogen *P. aeruginosa*, and could represent a target for eradication of recalcitrant infections *in situ*.

## Introduction

*Pseudomonas aeruginosa* is a highly adaptable opportunistic pathogen that is most commonly associated with severe, often fatal, nosocomial infections of the bloodstream (Tschudin-Sutter et al., 2018), urinary tract (Ferreiro et al., 2017) and upper respiratory tract of cystic fibrosis (CF) patients (Winstanley et al., 2016). Eradication of *P. aeruginosa* has become very difficult due to its capacity to resist conventional antibiotic therapy through various intrinsic, acquired and adaptive mechanisms (Pang et al., 2019). Adaptive resistance is particularly troubling since it is not genetically encoded and is induced by a wide array of environmental stimuli including antibiotic exposure, pH stress, anaerobiosis, and starvation (Breidenstein et al., 2011). Importantly, adaptive resistance is distinct from tolerance and persistence, which are characterized by metabolic dormancy that enable bacterial survival but not growth in the presence of antibiotics (Brauner et al., 2016). In contrast, adaptively multi-drug resistant cells actively divide and emerge during social lifestyles such as swarming motility and biofilm formation (Moradali et al., 2017; Coleman et al., 2020a).

Swarming is a complex adaptation involving rapid surface motility in which groups of cells collectively move using flagella and type IV pili to propel themselves over semi-solid agar in the presence of a weak nitrogen source, assisted by production of surface-wetting rhamnolipids (Yeung et al., 2009). Swarming is involved in bacterial dissemination from localized infection sites to distal organs (Coleman et al., 2020b) and is associated with increased resistance to most conventional antibiotics including ciprofloxacin, tobramycin and tetracycline (Coleman et al., 2020a). Similarly, surface-attached matrix-encapsulated communities of cells called biofilms exhibit up to 1000-fold higher (adaptive) multiple-antibiotic resistance than their planktonic counterparts (Olivares et al., 2020). Due to their ubiquity in nature, biofilms are often considered to represent the preferred growth state for bacteria in the environment and are implicated in more than 60% of all infections, often leading to chronic, recurrent infections (Flemming et al., 2016). The molecular mechanisms underlying swarming- and biofilm-mediated multi-drug resistance are not fully understood but are known to be multifactorial and largely independent of canonical resistance mechanisms (Lai et al., 2009; Ciofu and Tolker-Nielsen, 2019; Coleman et al., 2020a). A better understanding of these mechanisms is warranted for development of novel treatment strategies that target adaptive processes and dismantle bacterial virulence *in vivo* (Alford et al., 2019).

Adding to their complexity, swarming and biofilm lifestyles exhibit plasticity driven by environmental and genetic determinants (Kollaran et al., 2019; Thöming et al., 2020). For example, *P. aeruginosa* swarming is conserved but distinct on various nitrogen sources independent of growth differences (Alford et al., 2020). These nitrogen sources include nitrate, which is abundant in the lungs of CF patients (up to 400 μM; Line et al., 2014), as well as glutamate, which is also abundant in CF sputum (Palmer et al., 2005) and several other anatomical niches. Similarly, antibiotic susceptibility is dependent on central metabolism and can be potentiated or inhibited by addition of dicarboxylates such as fumarate or glyoxylate, respectively (Meylan et al., 2017; Hall et al., 2019). Tight regulation of gene expression through two-component system signaling allows *P. aeruginosa* to rapidly respond to environmental heterogeneity, contributing to its adaptability (Kollaran et al., 2019). NtrBC is a two-component system that regulates swarming motility, biofilm formation and nitrogen metabolism as well as invasiveness and virulence in a murine model of high-density infection (Alford et al., 2020). Since the transcriptional profile of NtrB and NtrC mutants demonstrated that genes involved in ciprofloxacin resistance were differentially expressed, we hypothesized they would be more susceptible *in vitro*. While confirming this data *in vivo*, we observed that, in the absence of ciprofloxacin, NtrBC mutants colonized the respiratory tracts, but not the skin, of mice to a lesser extent than WT. We hypothesized this effect was due to different availability of nitrogen in tissues and explored the impact of nitrogenous species on biofilm formation *in vitro* to confirm this. Furthermore, we demonstrated that NtrBC regulated host-directed cytotoxicity, virulence factor production and macrophage-mediated uptake, which might also contribute to differential colonization of host tissues.

## Materials and Methods

### Tissue Culture, Bacterial Strains, and Growth Conditions

Bacterial strains and plasmids used in this study are described in **Table S1**. Overnight cultures were routinely maintained in Luria-Bertani (LB) broth prepared according to the manufacturer’s specifications (Thermo Scientific). Overnight and sub-cultures were incubated for no longer than 18 h at 37°C with shaking (250 rpm). Modified forms of basal medium (BM2; containing 62 mM potassium phosphate buffer (pH = 7.0), 0.1% casamino acids (CAA), 2 mM MgSO_4_, 10 μM FeSO_4_, 20 mM glucose) were used for biofilm assays, kill curves and minimal inhibitory concentration (MIC) assays. For testing the influence of nitrogen on biofilm formation and ciprofloxacin killing, equimolar concentrations of nitrate $\left(\text{N}{\text{O}}_{3}^{-}\right)$, nitrite $\left(\text{N}{\text{O}}_{2}^{-}\right)$, glutamate (Glu) or urea replaced 0.1% casamino acids (CAA). Other media used in specific assays are described elsewhere. For plasmid selection in *P. aeruginosa* WT strains PA14 and LESB58, 50 μg/ml and 500 μg/ml gentamicin (Gm) was added to growth media. Bacterial growth was monitored by measuring optical density (OD_600_) with a spectrophotometer (Eppendorf, Missisauga, ON).

The Simian virus 40 (SV40)-transformed, immortalized human bronchial epithelial cell line 16HBE14o− (HBE) was used for the cytotoxicity assay. HBE cells were cultured in minimum essential medium with Earle’s Salts (MEM) supplemented with 10% fetal bovine serum (FBS, Gibco) and 2 mM L-glutamine (Gibco). The cell line was routinely cultured to 85 to 90% confluence in 100% humidity and 5% CO_2_ at 37°C and used between passages 9-15.

The human monocytic-like cell line THP-1 was obtained from the American Type Culture Collection (ATCC) and was routinely cultured in RPMI-1640 supplemented with 2 mM L-glutamine and 10% heat-inactivated FBS. Cells were differentiated into mature macrophages by stimulation with 100 ng/ml phorbol-12-myristate-13-acetate (PMA [P1585; Sigma]) for 48 h and then replaced with fresh medium without PMA for 24 h prior to the assay. The cell line was routinely cultured to 85 to 90% confluence in 100% humidity and 5% CO2 at 37°C.

### Minimal Inhibitory Concentration Assays

Broth microdilution assays were performed according to the standard protocol outlined by the Clinical and Laboratory Standards Institute (CLSI, 2020) with minor modifications. Bacteria were seeded at ~10^5^ CFU/ml in a 2-fold concentration gradient of antibiotic in Mueller-Hinton Broth (MHB) or BM2 with N-source, as indicated, at 200 μl/well in 96-well polystyrene flat bottom plates. Plates were incubated for 18 h at 37°C. MIC of antibiotics was determined as the lowest concentration that visibly inhibited bacterial growth.

### Kill Curves

Overnight cultures were diluted to a starting OD_600_ = 0.1 in 5 ml MHB. Cultures were grown to mid-log phase (OD_600_ = 0.4-0.6) at 37°C with aeration, and then treated with a high concentration of ciprofloxacin (25 μg/ml). Aliquots were taken every 30 or 60 min for up to 240 min following inoculation, diluted in PBS, pH 7.4 and plated on LB medium plates for bacterial enumeration.

### Study Approval and Animals

Animal experiments were performed in accordance with the Canadian Council on Animal Care (CCAC) guidelines and were approved by the University of British Columbia Animal Care Committee (protocol A17-0253). Mice used in this study were outbred C57Bl/6 mice (female, aged 11-13 weeks) or CD-1 mice (female, aged 5-7 weeks). All animals were purchased from Charles River Laboratories, Inc. (Wilmington, MA). C57Bl/6 and CD-1 mice weighed 20 ± 5 g and 25 ± 5 g, respectively, at the time of experiment and were group housed in cohorts of 4-5 littermates exposed to the same bacterial strain. Littermates were randomly assigned to experimental groups. Standard animal husbandry protocols were employed.

### Subcutaneous (Abscess) Infection

Ciprofloxacin susceptibility of LESB58 WT and mutants was assessed *in vivo* using a nuanced subcutaneous abscess model, as previously described (Pletzer et al., 2017). Bacterial cultures were grown to an OD_600_ of 1.0 in LB, washed twice in sterile PBS and resuspended to give a final inoculum of ~5 x 10^7^ CFU (in 50 μl). Bacteria were injected subcutaneously into the left dorsum of CD-1 mice and treated 1 h later with 50 μl of 0.2 mg/ml ciprofloxacin in endotoxin-free water. Abscesses were formed for 72 h, visible dermonecrosis was measured using a caliper at experimental endpoint and abscesses were harvested in PBS for bacterial enumeration on LB.

### Sinusitis Infection

Sinusitis infections were performed as previously described (Alford et al., 2021). Briefly, bacterial subcultures of *P. aeruginosa* LESB58 WT and mutant strains were washed twice with sterile PBS and resuspended at an OD_600_ of 1.0. Bacteria were instilled (20 μl), dropwise, into the left naris of C57Bl/6 mice under anesthesia (2.5% isoflurane) at 10^6^ CFU. At the experimental endpoint, mice were euthanized and nasal lavage as well as excision of lungs were performed for bacterial enumeration.

### Biofilm Formation

PA14 WT was examined for biofilm formation in the presence of different nitrogen (N)-sources using a high-throughput microtiter assay as described elsewhere (Fuente-Nunez et al., 2013). Overnight cultures were diluted to a starting OD_600_ = 0.1 in BM2 medium with 20 mM glucose and equimolar amounts of different N-sources and grown in polypropylene 96-well plates (Falcon). Following 18-24 h static incubation at 37°C, biomass was stained with 0.1% CV and dissolved in 70% ethanol. The OD_595_ was read using a BioTek SynergyH1 microplate reader (BioTek, Winooski, VT). Three independent experiments containing three biological replicates each were performed.

### Virulence Factor Assays

Pyoverdine was assessed as previously described (Imperi et al., 2009). Briefly, bacteria were incubated in Casamino acid medium (0.5% CAA, 0.1 mM MgSO_4_, 0.4% glucose, 7 mM potassium phosphate buffer, pH = 7.0) at 37°C (250 rpm). Turbid cultures were pelleted, and the supernatant was collected in a fresh microfuge tube. Five μl of supernatant was mixed with 995 μl 10 mM Tris-HCl (pH = 6.8). Fluorescence was measured at an excitation wavelength of 400 nm and emission 460 nm (Synergy H1 Microplate Reader, Biotek). Pyocyanin concentrations were determined spectrophotometrically after extraction with chloroform and 0.2 M HCl as described elsewhere (Essar et al., 1990). Absorbance at 520 nm was read (Synergy H1 Microplate Reader, Biotek). Elastase was determined by proteolysis of the Elastin-Congo red complex (Sigma) as described elsewhere (Ohman et al., 1980). Five hundred μl of supernatant from cultures grown for 16 h was collected, added to 10 mg/ml Elastin-Congo red in PBS (pH = 7.4) and incubated at 37°C (250 rpm) for 8 h. Absorbance of the aqueous fraction was examined at 495 nm (Synergy H1 Microplate Reader, Biotek).

### Toxicity Towards Human Bronchial Epithelial Cells

Confluent HBE cells were washed once with PBS, pH 7.4 (Gibco), removed by trypsinization with 0.25% Trypsin-EDTA (Gibco) and counted. HBE cells were seeded at ~7.5 x 10^5^ cells/well in 500 μl in a 24 well plate and grown again to confluency (2-3 days). Then, medium (MEM, 1% FBS, 2 mM L-glutamine) was refreshed and cells were rested for 1 h. Bacterial cultures grown to mid-log phase were pelleted, washed once with PBS and resuspended in medium. Bacterial cells were added to host cells at an MOI = 1, and co-cultures were maintained at 37°C with 5% CO_2_ for 12-16 h. HBE cell cytotoxicity was evaluated by measuring the release of lactate dehydrogenase (LDH) into the supernatant as previously described (Kumar et al., 2018).

### Macrophage Uptake (Gentamicin Protection Assay)

Macrophage-mediated uptake of PA14 WT and mutants was performed as described (Yeung et al., 2014) with minor modifications. Briefly, cultures were grown to mid-log phase (OD_600_ = 0.4-0.6), then washed with RPMI-1640 and resuspended in 1 ml of the medium. Mature macrophages were seeded in 24 well plates at ~3.5 × 10^5^ cells/well. Bacteria were added at an MOI of 10 and incubated for 1 h at 37°C since preliminary studies revealed that cytotoxicity was low at this point. Cells were washed with PBS and treated with 400 μg/ml gentamicin for 30 min at 37°C to remove residual bacteria from the well. Following treatment, macrophages were again washed, then left to rest for an additional 30-60 min or lysed with 0.1% Triton X-100. Macrophage lysate was plated onto LB agar for bacterial enumeration at 30, 60 or 90 min.

### Statistical Analysis

Statistics were performed using GraphPad Prism 8.0 (La Jolla, CA). *P* values were calculated as stated in the Figure captions. Statistical significance was established when *P* < 0.05.

## Results

### NtrBC Mutants Were More Sensitive to Ciprofloxacin

The two-component regulatory system NtrBC was recently shown to impact on the invasiveness of *P. aeruginosa* in an *in vivo* model as well as rhamnolipid synthesis, swarming motility and biofilm formation (Alford et al., 2020). We assessed whether it also directly impacted on susceptibility to antibiotics in MHB, a standard medium for determining MIC (**Table 1**). The MIC of the fluoroquinolone ciprofloxacin was 8-fold lower for Δ*ntrB* and Δ*ntrC* strains and ≥16-fold lower for Δ*ntrBC*. However, there were no significant differences in the MICs of tobramycin, chloramphenicol or tetracycline between *P. aeruginosa* PA14 strains.

**Table 1:** *P. aeruginosa* NtrBC mutants were more susceptible to ciprofloxacin.

Strain	Cip	Tb	Cap	Tc
**PA14 WT**	3.13	6.25	>200	1.56
**PA14 *ΔntrB***	0.39	6.25	>200	0.78
**PA14 *ΔntrC***	0.39	12.5	>200	1.56
**PA14 *ΔntrBC***	<0.39	6.25	>200	0.78

The minimal inhibitory concentration (MIC) of antibiotics was expressed as the lowest concentration that inhibited growth in at least two of three technical replicates in each of three independent experiments.

**Figure 1:**
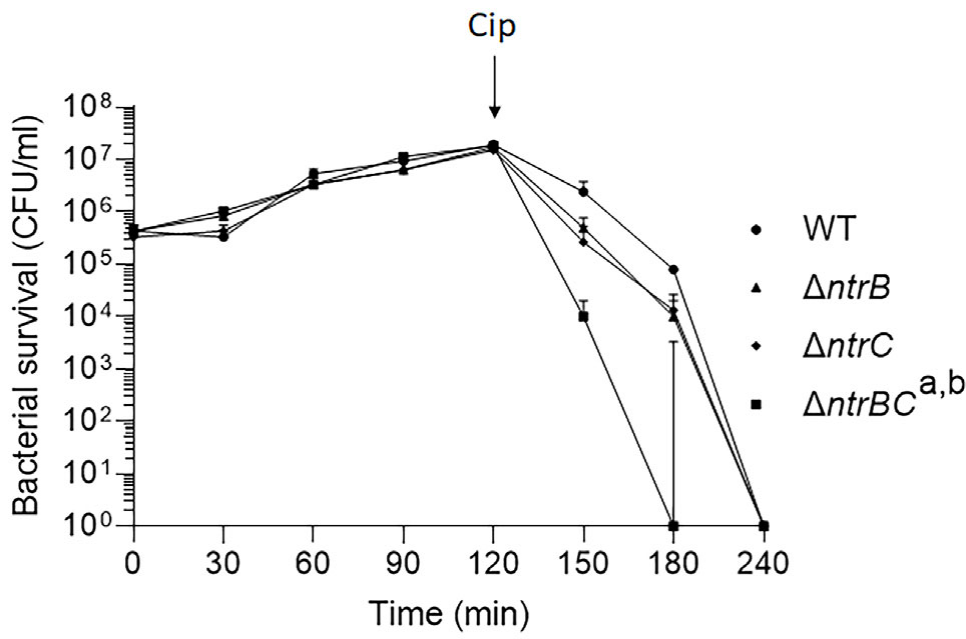
Mutation of the nitrogen regulatory two-component system, NtrBC, increased *P. aeruginosa* susceptibility to ciprofloxacin. Kill curves were performed in MHB after addition of 25 µg/ml ciprofloxacin at *t* = 120 min. Data are reported as mean ± standard error of the mean (SEM) from three independent experiments performed in duplicate. ^a,b^ *P* < 0.05 at t = 150 min and t = 180 min according to Kruskal Wallis nonparametric test followed by Dunn’s *post-hoc* analysis.

We then tested whether NtrBC directly impacted on the rate of killing of *P. aeruginosa* PA14 by treating mid-log phase cultures with a high concentration (25 µg/ml) of ciprofloxacin in MHB (**Figure 1**). Deletion of the entire two-component system (Δ*ntrBC*) resulted in significantly reduced viability by 2-log_10_ within 30 min of ciprofloxacin treatment and in most cases complete killing within 30-60 min of treatment, cf. WT that required 120 min for all bacteria to be killed. Deletion of just the response regulator (Δ*ntrB*) or the sensor kinase (Δ*ntrC*) from the chromosome of PA14 had no significant impact on the rate of killing. Consistent with this, N-source impacted on swarming-mediated resistance to ciprofloxacin (**Figure S1** and **Table S2**) exhibited by *P. aeruginosa*.

To explain the mechanism by which NtrBC influenced susceptibility to ciprofloxacin, we utilized published data (Alford et al., 2020) to examine the differential expression of genes in mutants belonging to the ciprofloxacin resistome (**Table 2**). Compared to WT, 17 of 287 ciprofloxacin resistome genes were downregulated in Δ*ntrB* and/or Δ*ntrC* strains.

**Table 2:** Genes in the ciprofloxacin resistome were downregulated in *P. aeruginosa* NtrBC mutants.

PA14 ID	Name	Annotation	FC Δ*ntrB*	FC Δ*ntr*C	Ref.^1^
PA14_03760	*gpuP*	3-guanidinopropionate transport protein		-4	1
PA14_10770	PA4112	sensor/response regulator hybrid		-1.8	1
PA14_13110	PA3924	probable medium-chain acyl-CoA ligase	-2.2	-2.8	1
PA14_18120	*mmsA*	methylmalonate-semialdehyde dehydrogenase	-1.9	-2.4	3
PA14_18830	PA3517	probable lyase	-2.7	-2.8	1
PA14_29420	PA2682	conserved hypothetical protein	-2.2	-2.7	3
PA14_29640	*fhp*	flavohemoprotein	-6.2	-5.8	3
PA14_33630	*pvdJ*	pyoverdine biosynthesis gene J	-2.4	-2.4	4
PA14_33650	*pvdD*	pyoverdine synthetase D	-2.1	-2.0	3
PA14_37310	PA2110	hypothetical protein	-8.4	-6.7	1
PA14_38690	PA1997	probable AMP-binding enzyme	-1.8	-1.6	2
PA14_45580	PA1459	probable methyltransferase	-1.7	-1.6	3
PA14_47100	*ilvA2*	threonine dehydratase, biosynthetic	-3.8	-3.5	1
PA14_47930	*lhpH*	4-hydroxyproline catabolism LhpH	-16.9	-9.7	1
PA14_53800	*mntH2*	manganese transport protein MntH		-2.3	1
PA14_55220	PA0703	MFS family transporter		-1.7	3
PA14_58690	PA4523	hypothetical protein	-1.5		3

^1^References 1 = Fajardo et al., 2008; 2 = Brazas et al., 2007; 3 = Breidenstein et al., 2008; 4= Dötsch et al., 2009.

Fastq and count files for all samples are available on the NCBI Gene Expression Omnibus (GEO) under accession number GSE145591. Analysis revealed differential expression of 17 genes belonging to the ciprofloxacin resistome that were significantly downregulated in NtrB and/or NtrC mutants relative to PA14 WT. Data are expressed as mean fold-change (FC) values from three biological replicates. Blank cells indicate no change in expression.

The most downregulated ciprofloxacin resistome gene was *lhpH*, the product of which is implicated in 4-hydroxyproline catabolism, that was expressed 16.9- and 9.7-fold less in Δ*ntrB* and Δ*ntrC* when compared to WT, respectively. Other substantially downregulated resistome genes encoded a putative allophanate hydrolase subunit with a carboxytransferase domain (PA14_37310/PA2110), a flavohemoprotein (*fhp*), a threonine dehydratase biosynthetic protein (*ilvA2*).

### NtrBC Mutants Were More Susceptible to Ciprofloxacin *In Vivo*

**Figure 2:**
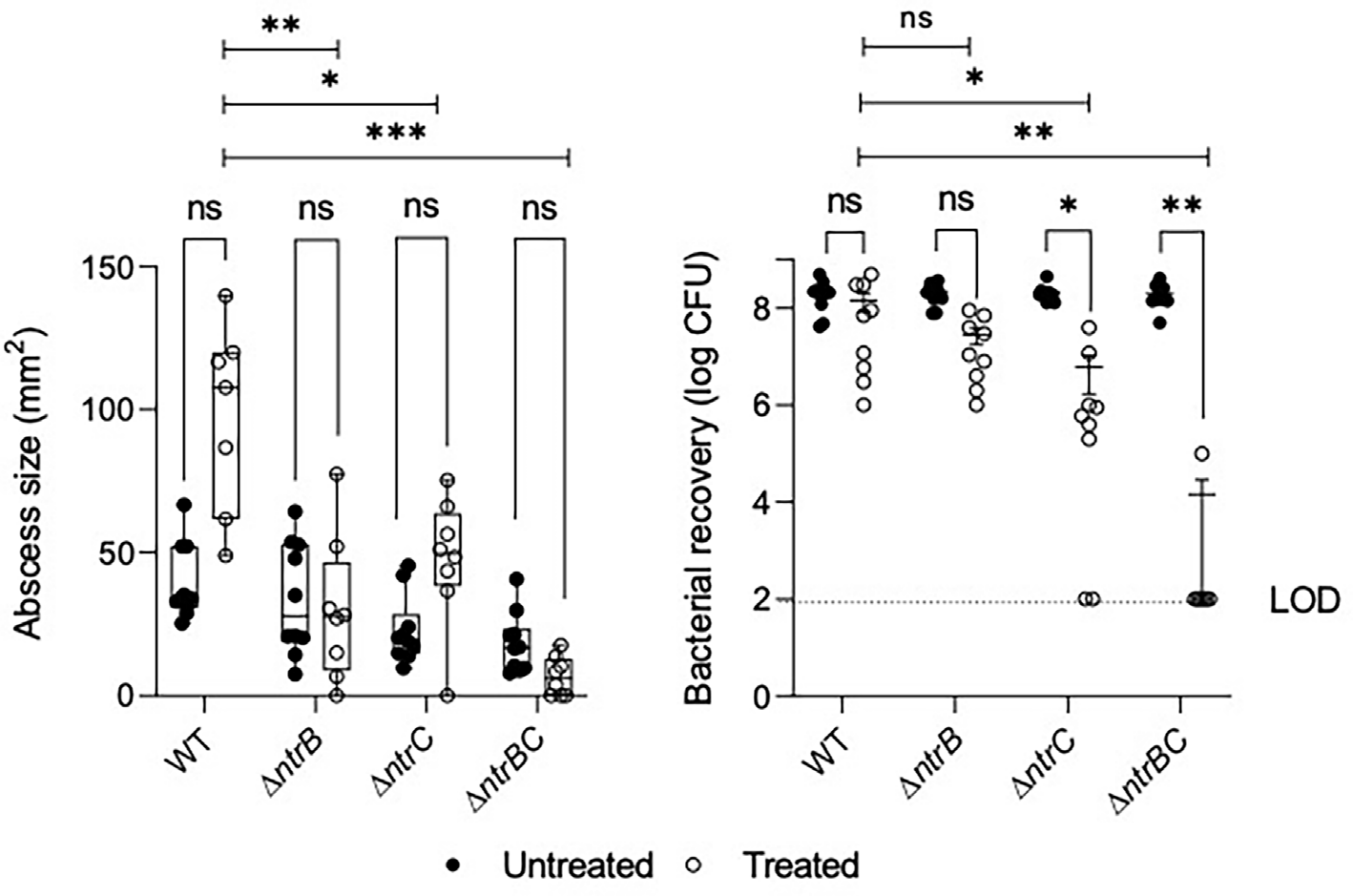
Mutation of the nitrogen regulatory two-component system, NtrBC, sensitized *P. aeruginosa* LESB58 to ciprofloxacin treatment *in vivo*. Abscesses formed by LESB58 Δ*ntrBC* following ciprofloxacin treatment (0.1 mg) were significantly smaller and contained fewer bacteria than those formed by WT. Briefly, mice were subcutaneously injected 5 x 10^7^ planktonic cells and treated 1 h later. After 72 h, abscesses were measured and harvested in phosphate buffered saline (PBS), homogenized and plated on LB for bacterial enumeration. Box and whiskers delineate interquartile range with geometric error from three independent experiments containing 2-3 biological replicates each (*n* = 8-10). Bacterial recovery data are reported as geometric mean ± standard deviation (SD). *P < 0.05, **P < 0.01, ***P < 0.001, ns, not significantly different compared to WT according to Two-Way ANOVA followed by Dunnett’s *post-hoc* analysis.

NtrBC was previously shown to be required for invasion in a murine abscess model of high-density infection (Alford et al., 2020). However, there were no differences in bacterial loads between *P. aeruginosa* LESB58 strains deleted for *ntrB*, *ntrC* or both. Since we observed that NtrBC is needed for resistance to ciprofloxacin *in vitro*, and ciprofloxacin was able to eradicate CF lung infection in a clinical trial (Hewer et al., 2020), we tested the ability of ciprofloxacin to kill these mutants *in vivo* (**Figure 2**). Treatment of WT with 0.1 mg ciprofloxacin did not significantly reduce abscess size when compared to untreated cells. Consistent with prior observations (Alford et al., 2020), deletion of *ntrB*, *ntrC* or both also failed to significantly reduce bacterial load compared to WT in the absence of treatment. However, mutants were more susceptible to ciprofloxacin *in vivo*. NtrBC mutants were reduced 4-log_10_ compared to WT following ciprofloxacin treatment.

Ciprofloxacin treatment also reduced bacterial load of LESB58 deletion mutants when compared to untreated cells of the same strain. In contrast, abscesses formed by LESB58 Δ*ntrBC* after ciprofloxacin treatment contained an average of only 1.4 x 10^4^ CFU (with most mice being completely cured), even though the Δ*ntrBC* deletion did not affect counts in the absence of ciprofloxacin treatment (2.0 x 10^8^ CFU recovered) compared to WT. Abscesses formed by LESB58 Δ*ntrC* exhibited a more moderate 30-fold decrease in bacterial load from 1.8 x 10^8^ CFU to 6.1 x 10^6^ CFU following ciprofloxacin treatment, while ciprofloxacin did not have a significant effect on LESB58 Δ*ntrB*.

### NtrBC Mutants Were Reduced for Respiratory Tract Colonization

**Figure 3:**
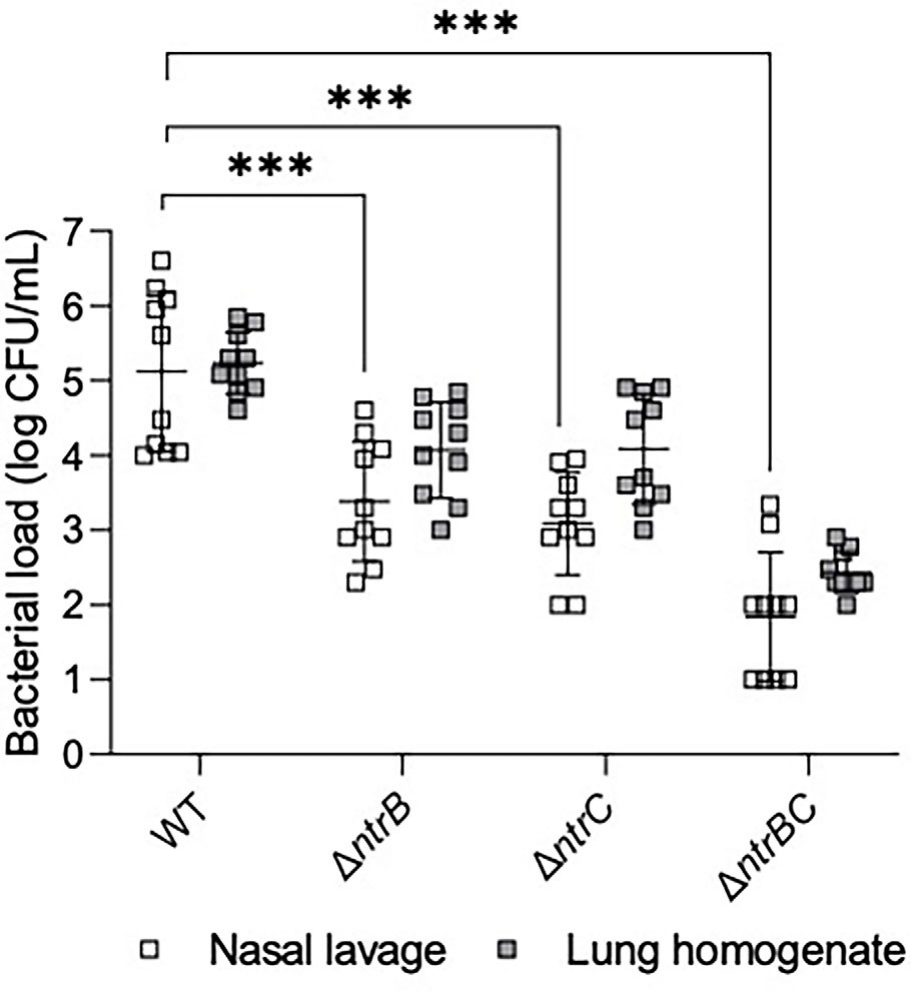
Mutation of the nitrogen regulatory two-component system, NtrBC, reduced bacterial load of *P. aeruginosa* in a murine model of sinusitis. Stationary-phase bacteria were inoculated dropwise in the left naris of C57Bl/6 mice (10^6^ CFU). 72 h later mice were euthanized, and lung tissue or nasal lavage fluid was collected for bacterial enumeration following serial dilution. Significantly less bacteria were recovered from the lungs and nasal cavities of mice infected with *P. aeruginosa* Δ*ntrB*, Δ*ntrC* or Δ*ntrBC* mutants. Data are presented as geometric mean ± standard deviation for three independent experiments containing 3-4 biological replicates each (*n* = 10). ***P < 0.001 according to Two-Way ANOVA followed by Dunnett’s *post-hoc* analysis.

The abscess model described above involves inoculation with a high density inoculum to create a chronic infection. We wondered whether the lack of effect of *ntrBC* deletion on abscess size or bacterial numbers would also hold in an acute model with lower input bacterial inocula. To investigate this, *P. aeruginosa* LESB58 strains were tested for colonization and virulence in the upper respiratory tract of mice (**Figure 3**) using our recently described airway/sinus infection model (Alford et al., 2021). In this model we determined that *ntrBC* deletion had a very substantial and significant effect when compared to WT, with significantly fewer Δ*ntrB*, Δ*ntrC* or Δ*ntrBC* isolated from the nasal cavity and lungs of mice, 72 h post-infection. Thus 96- to 298-fold fewer bacteria were isolated from the nasal cavity, while 8.0- to 10-fold less bacteria were found in the lungs of mice, infected with LESB58 Δ*ntrB* or Δ*ntrC* mutants. Moreover, the LESB58 Δ*ntrBC* double mutant demonstrated an even more profound effect with 2,155-fold and 766-fold fewer bacteria isolated from the nasal cavity and lungs respectively, although the latter was not significant. Conversely, no significant differences were detected for bacterial load of the same strain between nasal cavity and lungs, suggesting that these differences were consistent in these different areas of the respiratory tract. Importantly, *ntrBC* mutation reduced *P. aeruginosa* LESB58 colonization of the respiratory tract to a far greater extent than was observed in the high-density skin abscess model. This effect could be reversed by complementation of the mutants with the cloned *ntrBC* coding region (**Figure S2**).

### Effect of Nitrogen Source on Biofilm Formation in ESKAPE Pathogens

**Figure 4:**
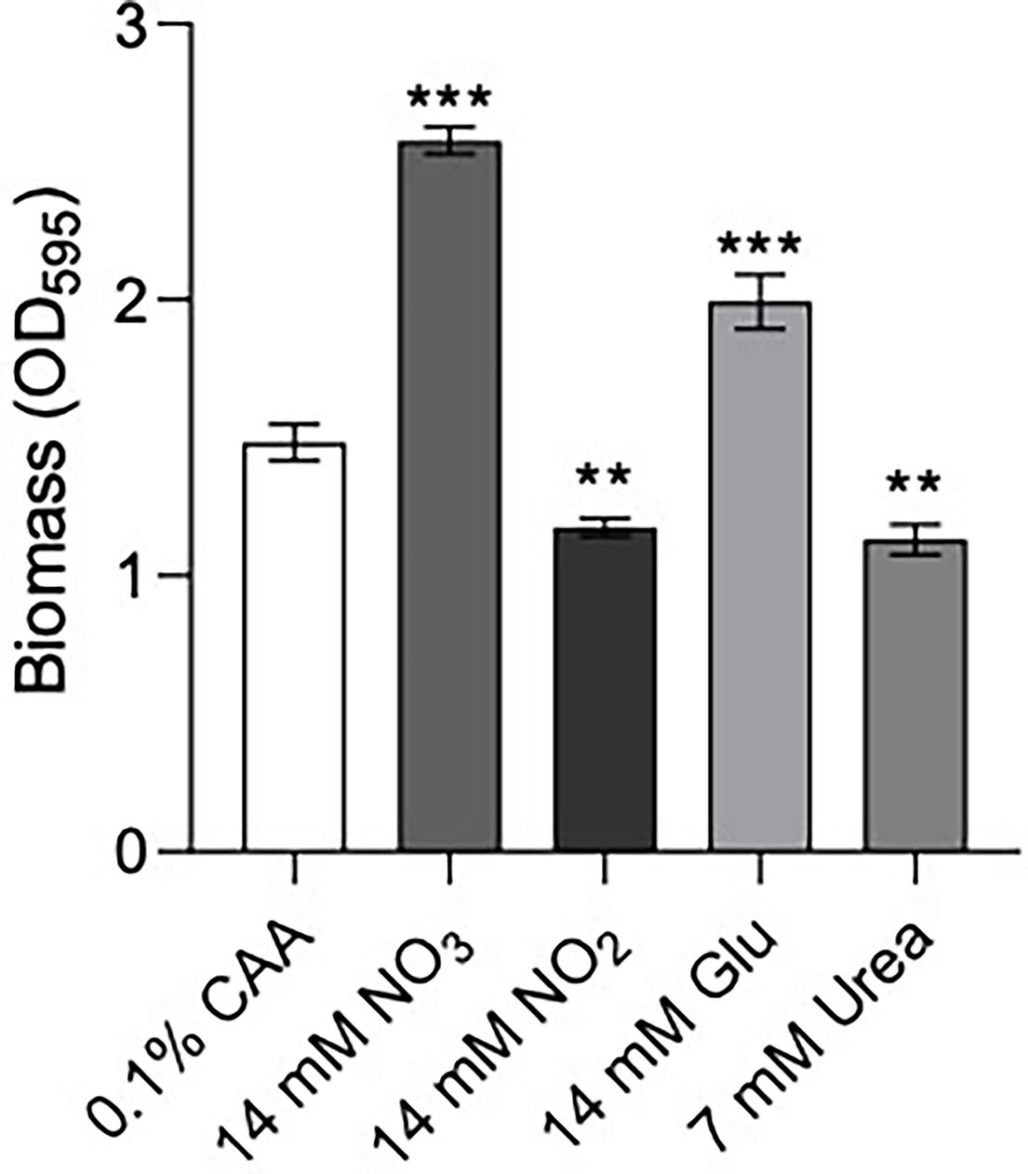
Biofilm formation of *P. aeruginosa* PA14 was influenced by nitrogen source. Biofilm was formed in BM2 medium containing casamino acids (CAA), nitrate $\left(\text{N}{\text{O}}_{3}^{-}\right)$, nitrite $\left(\text{N}{\text{O}}_{2}^{-}\right)$ glutamate (Glu), or urea in equimolar amounts. 96-well polypropylene plates were inoculated with bacteria suspended in basal medium (BM2) supplemented with 0.4% glucose. 18-20 h later, biomass was stained with crystal violet and measured by scanning its OD_595_. Data reported as mean ± standard error of the mean (SEM) from three independent experiments containing 2-3 biological replicates each (*n* = 6-9). **P < 0.01, ***P < 0.001 according to Kruskal-Wallis nonparametric test followed by Dunn’s *post-hoc* analysis.

We hypothesized that the influence of site of infection on NtrBC effects on colonization might reflect different N-sources at these sites. For example, it has been proposed that urea might be the predominant N-source in skin (Grice and Segre, 2011), while amino acids are the major N-source in the lung (Palmer et al., 2005). Thus, we examined the influence of N-source on biofilm formation (**Figure 4**), which is important for bacterial survival during chronic infection, and susceptibility of *P. aeruginosa* PA14 to ciprofloxacin killing *in vitro*.

Previous data suggested that Δ*ntrBC* but not Δ*ntrB*, or Δ*ntrC* alone had a substantial impact on biofilm formation (Alford et al., 2020). Therefore, we examined biofilm formation of *P. aeruginosa* PA14 in N-sources characteristic of different bodily niches, including casamino acids (CAA), nitrate $\left(\text{N}{\text{O}}_{3}^{-}\right)$, nitrite $(\text{N}{\text{O}}_{2}^{-})$, glutamate (Glu) and urea (**Figure 4**). Relative to that in CAA, biofilm biomass staining (OD_595_) was significantly increased to 174% and 130% when $\text{N}{\text{O}}_{3}^{-}$ and Glu replaced CAA in the growth medium. In contrast, when $\text{N}{\text{O}}_{2}^{-}$ or Urea replaced CAA, OD_595_ was decreased to 79.2% and 76.3% respectively.

### NtrBC Mutants Exhibited Reduced Production of Virulence Factors

**Figure 5:**
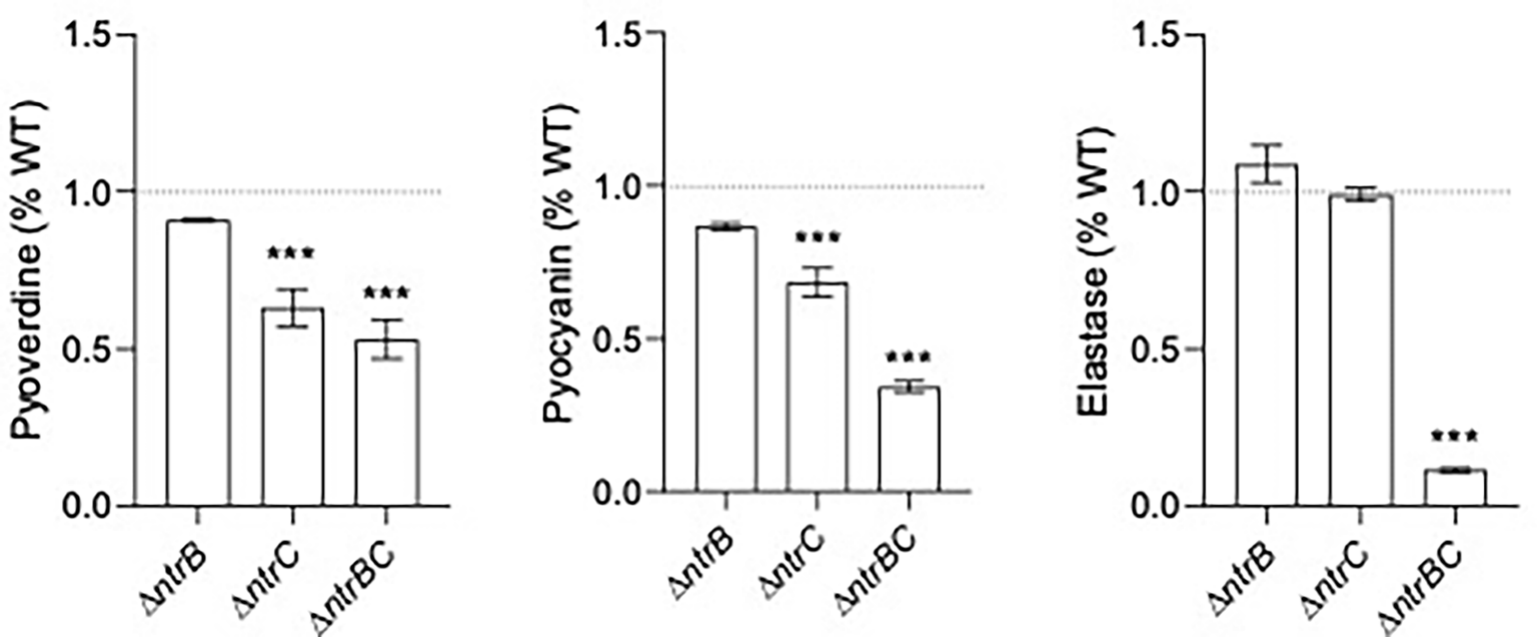
Mutation of the nitrogen regulatory two-component system, NtrBC, reduced production of virulence factors by *P. aeruginosa*. The supernatants of overnight cultures of similar density were assessed for pyoverdine (Pvd), pyocyanin (Pcn) and elastase (Las). Data were reported as mean ± standard error of the mean (SEM) from three independent experiments each containing three biological replicates (*n* = 9). ***P < 0.001 different from WT according to One-Way ANOVA followed by Dunn’s *post-hoc* analysis.

Since pyoverdine synthesis genes were expressed by 2.0- to 2.5-fold less in Δ*ntrB* and Δ*ntrC* strains compared to WT (**Table 2** and **Table S3**), and because mutants were less capable of colonizing the respiratory tract, we measured production of virulence factors including pyoverdine, pyocyanin and elastase by PA14 strains grown overnight for 16 hours (**Figure 5**). Although there were no statistically significant differences between PA14 WT and Δ*ntrB*, production of pyoverdine and pyocyanin in Δ*ntrC* was significantly reduced by 38.2% and 31.7%, respectively. Production of all virulence factors was significantly and substantially reduced in the Δ*ntrBC* double mutant, by 48.2%, 65.2% and 89.8% in pyoverdine, pyocyanin and elastase respectively. This coincided with the downregulated expression of 19 genes involved in the production of siderophores or phenazines in Δ*ntrB* and/or Δ*ntrC* (**Table S3**). Substantial reductions in virulence determinants might be expected to reduce the ability to colonize.

### NtrBC Mutants Elicited More Robust Host-Cell Responses

**Figure 6:**
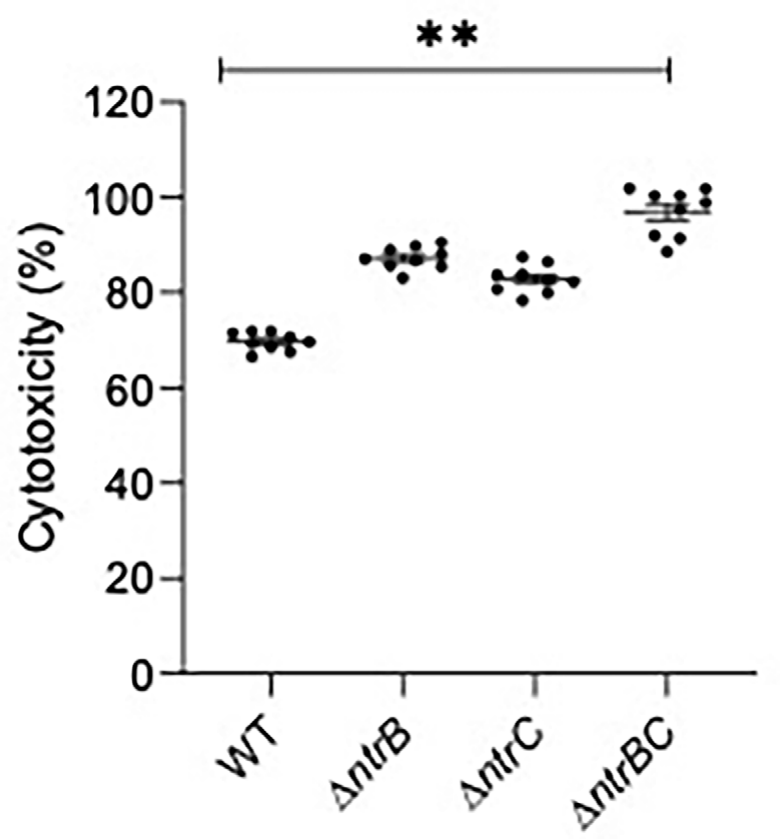
Mutation of the nitrogen regulatory two-component system, NtrBC, increased host-directed cytotoxicity of *P. aeruginosa*. Confluent human bronchial epithelial (HBE) cells (~7.5 x 10^4^ cells/well) were treated with mid-log phase bacteria at a multiplicity of infection (MOI) = 1 and incubated at 37°C for 16-18 h. Cell-free supernatants were collected and host-directed cytotoxicity (%) was estimated by LDH release from cells relative to cells lysed with Triton X-100. Significantly more cytotoxicity was caused after infection with *P. aeruginosa* Δ*ntrBC* mutants. Data are presented as mean ± standard error of the mean from four independent experiments containing three biological replicates each (*n* = 12). **P < 0.01 according to One-Way ANOVA followed by Dunn’s *post-hoc* analysis.

Since virulence factor production is a key determinant of *P. aeruginosa* pathogenesis (Alford et al., 2019, Strateva and Mitov, 2011), and NtrBC directly impacted on the levels of secreted virulence factors including cytotoxins like elastase (**Figure 5**) and rhamnolipids (Alford et al., 2020), we studied whether NtrBC modulated host-directed cytotoxicity in response to *in vitro* infection (**Figure 6**). A modest but non-significant increase in cytotoxicity of HBE cells was observed following infection with Δ*ntrB* or Δ*ntrC* (13.2% and 7.03% greater than WT, respectively) at an MOI = 1, and a modest but significant 21.5% increase in cytotoxicity was observed following infection with Δ*ntrBC*.

**Figure 7:**
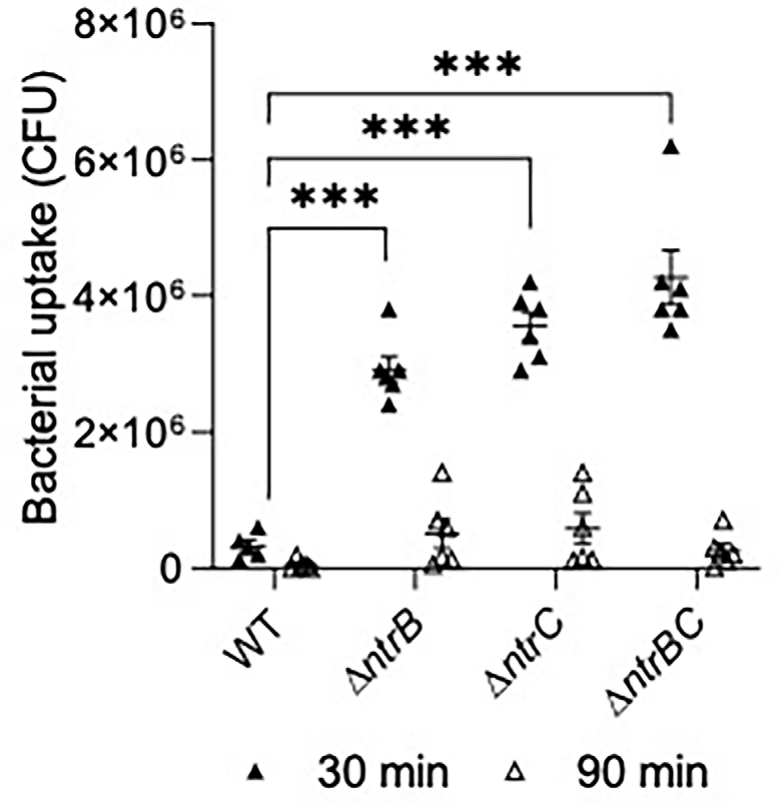
Mutation of the nitrogen regulatory two-component system, NtrBC, increased uptake of *P. aeruginosa* by macrophages. Confluent THP-1 monocytes (~3.0 x 10^5^ cells/well) were differentiated and treated with mid-log phase bacteria at a multiplicity of infection (MOI) = 10 and incubated at 37°C for 30 min. Cells were washed, treated with gentamicin (500 µg/ml) for 30 min, and washed again to remove any residual bacteria in the supernatant. Cell lysates at t = 0, 60 min were plated for bacterial enumeration following serial dilution. Mutants were taken up and cleared from macrophages more efficiently than the WT. Data are presented as geometric mean ± standard deviation for three independent experiments containing 2 biological replicates each (*n* = 6). ns, not statistically different; ***P < 0.001 different according to Two-way ANOVA followed by Dunnett’s *post-hoc* analysis.

We next tested susceptibility to nonopsonic phagocytosis by examining macrophage-mediated uptake and clearance of pathogens at an MOI = 10 (**Figure 7**). Using a gentamicin-protection assay to record bacteria taken up by macrophages, lysates from infections with Δ*ntrB*, Δ*ntrC* or Δ*ntrBC* contained 9.0- to 13.2-fold more bacteria than lysates from infections with WT at 30 min, indicating substantially greater uptake of these mutants. This coincided with significant upregulation of 38 genes important for macrophage uptake (Felgner et al., 2020) (**Table S3**). There were no statistically significant differences between mutants and WT at 90 min. Nonetheless, each strain was reduced, on average, by ~10-fold at the later time point, indicating similar rates of clearance. In contrast, pilot studies showed that neutrophil-mediated uptake of PA14 WT and mutants were not significantly different (**Figure S3**).

## Discussion and Conclusion

NtrBC is a two-component system of *P. aeruginosa* that is essential for sensing environmental nitrogen levels and responding to nitrogen starvation through initiation of nitrate assimilation (Luque-Almagro et al., 2011). NtrC is known to activate a variety of other physiological processes, in part by increasing the binding affinity of the alternative sigma factor RpoN/σ^54^ to RNA polymerase, including histidine utilization (Naren and Zhang, 2021) and the stringent stress response (Brown et al., 2014). We previously showed that NtrBC impacts on *P. aeruginosa* adaptive phenotypes including biofilm formation and swarming motility as well as invasiveness and virulence *in vivo* (Alford et al., 2020), and that the effects of each of NtrB and NtrC appear to be additive for selected phenotypes. The results described here are distinct from those shown previously, since we demonstrated that NtrBC regulated colonization in a tissue-specific manner by affecting certain host-pathogen interactions and production of virulence factors. We also explored the role of nitrogen in adaptive phenotypes, including biofilm formation and swarming-mediated antibiotic resistance.

Adding to our knowledge of NtrBC as a global regulator, the results shown here indicated that activity of this two-component system directly impacted on ciprofloxacin resistance of *P. aeruginosa* (**Figure 1** and **Table 1**) without influencing susceptibility to tobramycin, tetracycline or chloramphenicol. This was recapitulated in an abscess model of high-density infection (**Figure 2**). Ciprofloxacin is one of the most important antibiotics used for the treatment of CF lung infections of both children and adults (Andriole, 2005). However, ciprofloxacin resistance is on the rise, with 40% of CF isolates sampled in one study exhibiting resistance (Landman et al., 2007). Ciprofloxacin resistance is usually multifactorial, involving expulsion by the multidrug efflux-porin systems (Rehman et al., 2019), as well as target site mutation in the DNA gyrase (*gyrAB*) or topoisomerase (*parCE*) that are targeted by ciprofloxacin (Feng et al., 2019). We identified other, non-canonical effectors of ciprofloxacin resistance downstream of NtrBC that spanned multiple physiological categories (**Table 1**). Some of the most significantly dysregulated effectors included the intracellular protease PfpI (Fernández et al., 2012), which has been shown to affect swarming and biofilm formation as well as resistance, the acyl-coA ligase PA3924 (Zarzycki-Siek et al., 2013), which is needed for nutrient acquisition in the murine lung, flavohemoprotein fhp (Koskenkorva-Frank and Kallio, 2009), which is induced to protect against nitrosative stress, and catabolic protein LhpH (Li and Lu, 2016), which is implicated in use of the major non-proteinogenic amino acid in mammals, hydroxyproline. Clearly, NtrBC is a regulatory system at the intersection of ciprofloxacin resistance and metabolic processes of *P. aeruginosa*.

As a metabolically versatile organism with a large genome comprising numerous regulatory systems, *P. aeruginosa* is able to establish chronic, recurrent infections in various niches of the human body (Frimmersdorf et al., 2010). In the airways of CF patients, *P. aeruginosa* undergoes a broad metabolic rewiring (Bartell et al., 2017; La Rosa et al., 2019) to accommodate the needs of the cell according to diversity and availability of nutrients, including free amino acids and $\text{N}{\text{O}}_{3}^{-}$ that are not detected in abundance in other bodily niches (Palmer et al., 2005). Thus, interference with metabolism of these nitrogen sources could inhibit colonization ability in a tissue-specific manner. In support, we showed that *P. aeruginosa* LESB58 Δ*ntrB* and/or Δ*ntrC* mutants, which are deficient for metabolism of nitrogenous species including $\text{N}{\text{O}}_{3}^{-}$ (Alford et al., 2020) and Glu, but not urea, colonized the skin to a similar extent as WT in the absence of ciprofloxacin treatment, but did not colonize the respiratory tract of mice as well as WT (**Figure 3**). This effect could be mitigated by complementation of the *ntrBC* coding region back into mutants (**Figure S2**). This contributes to our understanding of the complex physiological processes assumed by bacteria during infection that impacts pathogenesis and antimicrobial intervention (Alford et al., 2019) and indicates that NtrBC is important for metabolic adaptation in the nasal cavity and lungs, but not the skin. This data also implicates NtrBC in recurrent CF infection, since *P. aeruginosa* may persist in the sinus cavity of patients, representing a reservoir where it can adapt and disseminate into the lower respiratory tract over time (Fothergill et al., 2014). As well, increased susceptibility to ciprofloxacin was conferred by the LESB58 Δ*ntrB* and/or Δ*ntrC* mutation *in vivo* (**Figure 2**), validating *in vitro* MIC results in physiologically relevant conditions, which is considered meaningful since bacterial susceptibility to antimicrobials is heavily dependent on environmental conditions.

Indeed, pathoadaptivity and metabolism are intrinsically associated, and nutritional cues in CF sputum impact on multicellular behaviors such as biofilm formation and swarming motility (Palmer et al., 2007). We previously showed that N-source impacted on swarming motility of *P. aeruginosa* (Alford et al., 2020) and that certain nitrogen-containing compounds, including ammonium and urea, did not support swarming motility as did CAA and $\text{N}{\text{O}}_{3}^{-}$. Here we provide further evidence showing that N-source impacts on biofilm formation (**Figure 4**), and that the effects on biofilm and swarming phenotypes are correlated, which taken together could partially explain the distinct tissue colonization patterns of *P. aeruginosa* LESB58 strains. More specifically, the nitrogen sources that promoted biofilm formation and swarming ($\text{N}{\text{O}}_{3}^{-}$ and the free amino acid Glu, relative to CAA) are those that are abundant in the respiratory tract but not the skin (Palmer et al., 2005; Grice and Segre, 2011). Further, Δ*ntrB* and/or Δ*ntrC* mutants do not grow well in these N-sources, nor do they undergo biofilm formation or swarming motility as well as WT in BM2 containing CAA as the N-source (Alford et al., 2020). Thus, there is the potential that other adaptive species are outcompeting Δ*ntrBC* mutants for the respiratory tract. Furthermore, N-source influenced the ability of *P. aeruginosa* to swarm in the presence of a high concentration of ciprofloxacin (**Figure S1** and **Table S2**). At ~8x its MIC, ciprofloxacin inhibited swarming of PA14 on BM2 containing CAA as the N-source. However, swarming was still observed in the presence of this concentration of ciprofloxacin following substitution of CAA for Glu and NO3^-^. Thus, PA14 exhibited swarming-associated resistance to ciprofloxacin in a nitrogen-dependent manner. This data might have implications for the development of metabolism-based therapeutics. For instance, compounds that antagonize $\text{N}{\text{O}}_{3}^{-}$ and Glu metabolism may be explored as possible therapies to prevent *P. aeruginosa* adaptations *in situ* since provision of $\text{N}{\text{O}}_{3}^{-}$ and Glu as the sole N-source promoted adaptive phenotypes across the board. This data shows the importance of considering experimental conditions and bacterial lifestyle when determining the activity of emerging antibacterial compounds (Alford et al., 2019). Further, swarming-mediated resistance of bacteria to several clinically important antibiotics was conserved across motile species including *Salmonella enterica* and Gram-positive *Bacillus subtilis* (**Table S2**). Thus, therapies targeting adaptive lifestyles of bacteria could be developed as broad-spectrum antimicrobial agents with potential for limiting bacterial dissemination *in vivo* (Coleman et al., 2020b).

Since genes implicated in production of pyoverdine (*pvdD*, *pvdJ*) were also significantly downregulated in Δ*ntrB* and Δ*ntrC* mutants (**Tables 2** and **S3**), we assessed PA14 strains for their production of virulence factors (**Figure 5**). It was shown that strains defective for nitrogen metabolism produced less pyocyanin, pyoverdine and elastase. Although pathoadapted strains of *P. aeruginosa* isolated from chronic CF lung infections are characterized by reduced virulence overall, a recent study identified significant within-patient phenotypic diversity of isolates (O’Brien et al., 2017) characterized by co-existence of strains that under-produce or overproduce pyoverdine and elastase. This might be caused by different availability of nutrients in niches of the lung, since virulence factor production is associated with core metabolic activity of *P. aeruginosa* (Panayidou et al., 2020) and is inhibited following perturbation of metabolism during biofilm formation (Perinbam et al., 2020). These observations align with what we observed herein.

Though virulence factor production was downregulated, we noted significantly more cytotoxicity toward HBE cells by the PA14 Δ*ntrBC* mutant strain than the WT (**Figure 6**). The first step in establishing *P. aeruginosa* infection of the airway is receptor-mediated binding to the apical surface of the epithelium, which depends on type IV pili and the type III secretion system (Bucior et al., 2014). Type IV pili are functional in the PA14 Δ*ntrB*, Δ*ntrC* and Δ*ntrBC* strains since they twitched normally (Alford et al., 2020). Similarly, the type III secretion system is upregulated in mutants compared to WT (**Table S3**). Indeed, expression of the type III apparatus encoded by *pscBCEP* (Yang et al., 2007) is upregulated 3.0- to 7.5-fold in Δ*ntrB* or Δ*ntrC* when compared to WT (Alford et al., 2020), and expression of type III effectors encoded by *pcr1* and *pcr3* are upregulated 6.1-fold and 12-fold, respectively (Alford et al., 2020). However, since we observed that PA14 mutants were less virulent in the skin abscess model (i.e. caused less visible dermonecrosis even in the absence of ciprofloxacin), we were surprised by these results. Innate immunity mediated by phagocytic clearance by neutrophils and macrophages is key to the endogenous control of *P. aeruginosa* (Lovewell et al., 2014) and may also explain prior observations that Δ*ntrBC* mutants was less invasive (Alford et al., 2020). Therefore, we studied whether uptake by macrophages was also affected by mutation of this regulatory system (**Figure 7**). Indeed Δ*ntrB*, Δ*ntrC* and Δ*ntrBC* mutants were more susceptible to phagocytosis by mature macrophages, but demonstrated similar clearance rates (**Figure 7**) when compared to PA14 WT. In this regard it is worth noting that *ntrBC* mutants have functional flagella and swim and twitch normally (Alford et al., 2020) given that both flagella and pili are involved in non-opsonic phagocytosis by macrophages (Kelly et al., 1989; Lovewell et al., 2014). Production of type III toxins such as *exoSTY* has been shown to limit uptake of *P. aeruginosa* by macrophages (Hauser, 2009), and the expression of these is modestly downregulated in *ntrBC* mutants. Type III toxin (*exoSTY*) secretion by mutants should be assessed in future studies, particularly since genes encoding type III apparatus (*pscBEP*) and other effectors (*pcr1* and *pcr3*) exhibited opposite expression.

Despite extensive studies on the physiology of *P. aeruginosa*, we have only a limited understanding of the relationship between core metabolism (including nitrogen assimilation) and adaptive resistance or virulence, particularly in pre-clinical disease models. This study shows a clear role for nitrogen metabolism and NtrBC regulation in ciprofloxacin susceptibility and virulence attributes of *P. aeruginosa* and suggests NtrBC as a potential target for the development of future metabolism-directed or virulence-attenuating therapeutics. Further studies should be performed to elucidate the interacting partners of NtrB and NtrC that could be contributing to their apparent dose-dependent influence on certain phenotypes and to predict compensatory mechanisms that could be triggered by inhibition of NtrB and NtrC *in situ*.

## Data Availability Statement

The raw data supporting the conclusions of this article will be made available by the authors, without undue reservation.

## Ethics Statement

The animal study was reviewed and approved by the Canadian Council on Animal Care (CCAC) guidelines and were approved by the University of British Columbia Animal Care Committee (protocol A17-0253).

## Author Contributions

MA was responsible for investigation, validation and visualization of data, formal analysis, writing (drafting and editing) and project administration. BB, AA, and KY-C were responsible for experiments and writing (editing). RH was responsible for funding acquisition, provision of resources and supervision, project administration and writing (editing). All authors contributed to the article and approved the submitted version.

## Funding

We gratefully acknowledge funding to RH from the Canadian Institutes for Health Research grant FDN-154287 and Michael Smith Foundation for Health Research grant 17774. RH holds a Canada Research Chair in Health and Genomics and a UBC Killam Professorship. MA holds a UBC Killam Doctoral Scholarship, Four-Year Fellowship and CIHR Vanier Graduate Scholarship. AA also holds a UBC Killam Doctoral Scholarship while K-YC holds a Michael Smith Foundation for Health Research postdoctoral fellowship.

## Conflict of Interest

The authors declare that the research was conducted in the absence of any commercial or financial relationships that could be construed as a potential conflict of interest.

## References

[B1] Alford M. A. Baghela A. Yeung A. T. Y. Pletzer D. Hancock R. E. W. (2020). NtrBC Regulates Invasiveness and Virulence of *Pseudomonas aeruginosa* During High-Density Infection. Front. Microbiol. 11, 773. 10.3389/fmicb.2020.00773 32431676 PMC7214821

[B2] Alford M. A. Choi K. Y. G. Trimble M. J. Masoudi H. Kalsi P. Pletzer D.. (2021). Model of Sinusitis Infection for Screening Antimicrobial and Immunomodulatory Therapies. Front. Cell. Infect. Microbiol. 11, 621081. 10.3389/fcimb.2021.621081 33777834 PMC7994591

[B3] Alford M. A. Pletzer D. Hancock R. E. W. (2019). Dismantling the Bacterial Virulence Program. Microb. Biotechnol. 12 (3), 409–413. 10.1111/1751-7915.13388 30864265 PMC6465231

[B4] Andriole V. T. (2005). The Quinolones: Past, Present, and Future. Clin. Infect. Dis. 41 (2), S113–S119. 10.1086/428051 15942877

[B5] Bartell J. A. Blazier A. S. Yen P. Thørgensen J. C. Jelsbak L. Goldberg J. B.. (2017). Reconstruction of the Metabolic Network of *Pseudomonas aeruginosa* to Interrogate Virulence Factor Synthesis. Nat. Commun. 8:14631. 10.1038/ncomms14631 28266498 PMC5344303

[B6] Brauner A. Fridman O. Gefen O. Balaban N. Q. (2016). Distinguishing Between Resistance, Tolerance and Persistence to Antibiotic Treatment. Nat. Rev. Microbiol. 14, 320–330. 10.1038/nrmicro.2016.34 27080241

[B7] Brazas M. D. Breidenstein E. B. M. Overhage J. Hancock R. E. W. (2007). Role of Lon, An ATP-Dependent Protease Homolog, in Resistace of *Pseudomonas Aeruginosa* to Ciprofloxacin. Antimicrob. Agents Chemother. 51 (12), 4276–4283. 10.1128/AAC.00830-07 17893152 PMC2167996

[B8] Breidenstein E. B. M. Fuente-Nuñez C. Hancock R. E. W. (2011). *Pseudomonas Aeruginosa*: All Roads Lead to Resistance. Trends Microbiol. 19 (8), 419–426. 10.1016/j.tim.2011.04.005 21664819

[B9] Breidenstein E. B. M. Khaira B. K. Wiegand I. Overhage J. Hancock R. E. W. (2008). Complex Ciprofloxacin Resistome Revealed by Screening a *Pseudomonas Aeruginosa* Mutant Library for Altered Susceptibility. Antimicrob. Agents Chemother. 52 (12), 4486–4491. 10.1128/AAC.00222-08 18824609 PMC2592849

[B10] Brown D. R. Barton G. Pan Z. Buck M. Wigneshweraraj S. (2014). Nitrogen Stress Response and Stringent Response are Coupled in *Escherichia Coli* . Nat. Commun. 5, 4115. 10.1038/ncomms5115 24947454 PMC4066584

[B11] Bucior I. Tran C. Engel J. (2014). Assessing *Pseudomonas* Virulence Using Host Cells. Methods Mol. Biol. 1149, 741–755. 10.1007/978-1-4939-0473-0_57 24818947 PMC4083080

[B12] Ciofu O. Tolker-Nielsen T. (2019). Tolerance and Resistance of *Pseudomonas aeruginosa* Biofilms to Antimicrobial Agents - How *P. Aeruginosa* Can Escape Antibiotics. Front. Microbiol. 10, 913. 10.3389/fmicb/2019.00913 31130925 PMC6509751

[B13] Clinical and Laboratory Standards Institute (CLSI) (2020). Performance Standards for Antimicrobial Susceptibility Testing, 30th ed, Wayne, PA: Clinical and Laboratory Standards Institute. ISBN: 978-1-68440-067-6, CLSI supplement M100.

[B14] Coleman S. R. Blimkie T. Falsafi R. Hancock R. E. W. (2020a). Multi-Drug Adaptive Resistance of *Pseudomonas aeruginosa* Swarming Cells. Antimicrob. Agents Chemother. 64 (3), e01999–e01919. 10.1128/AAC.01999-19 31844008 PMC7038238

[B15] Coleman S. R. Pletzer D. Hancock R. E. W. (2020b). Contribution of Swarming Motility to Dissemination in a *Pseudomonas aeruginosa* Murine Skin Abscess Infection Model. J. Infect. Dis. jiaa778. 10.1093/infdis/jiaa778 33349847 PMC8366438

[B16] Dötsch A. Becker T. Pommerenke C. Magnowska Z. Jänsch L. Häussler S. (2009). Genomewide Identification of Genetic Determinants of Antimicrobial Drug Resistance in *Pseudomonas aeruginosa* . Antimicrob. Agents Chemother. 53 (6), 2522–2531. 10.1128/AAC.00035-09 19332674 PMC2687185

[B17] Essar D. W. Eberly L. Hadero A. Crawford I. P. (1990). Identification and Characterization of Genes for a Second Anthranilate Synthase in *Pseudomonas aeruginosa*: Interchangeability of the Two Anthranilate Synthases and Evolutionary Implications. J. Bacteriol. 172, 884–900. 10.1128/jb.172.2.884-900.1990 2153661 PMC208517

[B18] Fajardo A. Martínez-Martín N. Mercadillo M. Galán J. C. Ghysels B. Matthijs S.. (2008). The Neglected Instrinsic Resistome of Bacterial Pathogens. PLoS One 3 (2), e1619. 10.1371/journal.pone.0001619 18286176 PMC2238818

[B19] Felgner S. Preusse M. Beutling U. Stahnke S. Pawar V. Rohde M.. (2020). Host-Induced Spermidine Production in Motile *Pseudomonas aeruginosa* Triggers Phagocytic Uptake. eLife 9, e55744. 10.7554/eLife.55744 32960172 PMC7538158

[B20] Feng X. Zhang Z. Li X. Song Y. Kang J. Yin D.. (2019). Mutations in *gyrB* Play an Important Role in Ciprofloxacin-Resistant *Pseudomonas aeruginosa* . Infect. Drug Resist. 12, 261–272. 10.2147/idr.s182272 30804676 PMC6371945

[B21] Fernández L. Breidenstein E. B. M. Song D. Hancock R. E. W. (2012). Role of Intracellular Proteases in the Antibiotic Resistance, Motility and Biofilm Formation of *Pseudomonas aeruginosa* . Antimicrob. Agents Chemother. 56 (2), 1128–1132. 10.1128/AAC.05336-11 22123702 PMC3264200

[B22] Ferreiro J. L. L. Otero J. Á. González L. G. Lamazares L. N. Blanco A. A. Sajurjo J. R. B.. (2017). *Pseudomonas aeruginosa* Urinary Tract Infections in Hospitalized Patients: Mortality and Prognostic Factors. PLoS One 12 (5), e0178178. 10.1371/journal.pone.0178178 28552972 PMC5446154

[B23] Flemming H. Wingender J. Szewzyk U. Steinberg P. Rice S. A. Kjelleberg S. (2016). Biofilms: An Emergent Form of Bacterial Life. Nat. Rev. Microbiol. 14, 563–575. 10.1038/nrmicro.2016.94 27510863

[B24] Fothergill J. L. Neill D. R. Loman N. Winstanley C. Kadioglu A. (2014). *Pseudomonas aeruginosa* Adaptation in the Nasopharyngeal Reservoir Leads to Migration and Persistence in the Lungs. Nat. Commun. 5, 4780. 10.1038/ncomms5780 25179232

[B25] Frimmersdorf E. Horatzek S. Pelnikevich A. Wiehlmann L. Schomburg D. (2010). How *Pseudomonas aeruginosa* Adapts to Various Environments: A Metabolomic Approach. Environ. Microbiol. 12, 1734–1747. 10.1111/j.1462-2920.2010.02253.x 20553553

[B26] Fuente-Nunez C. Reffuveille F. Fernandez L. Hancock R. E. W. (2013). Bacterial Biofilm Development as a Multicellular Adaptation: Antibiotic Resistance and New Therapeutic Strategies. Curr. Opin. Microbiol. 16 (5), 580–589. 10.1016/j.mib.2013.06.013 23880136

[B27] Grice E. A. Segre J. A. (2011). The Skin Microbiome. Nat. Rev. Microbiol. 9 (4), 244–253. 10.1038/nrmicro2537 21407241 PMC3535073

[B28] Hall C. W. Farkas E. Zhang L. Mah T. F. (2019). Potentiation of Aminoglycoside Lethality by C_4_-dicarboxylates Requires RpoN in Antibiotic-Tolerant *Pseudomonas aeruginosa* . Antimicrob. Agents Chemother. 63 (10), e01313–e01319. 10.1128/AAC.01313-19 31383655 PMC6761562

[B29] Hauser A. R. (2009). The Type III Secretion System of *Pseudomonas aeruginosa*: Infection by Injection. Nat. Rev. Microbiol. 7 (9), 654–665. 10.1038/nrmicro2199 19680249 PMC2766515

[B30] Hewer S. C. L. Smyth A. R. Brown M. Jones A. P. Hickey H. Kenna D.. (2020). Intravenous Versus Oral Antibiotics for Eradication of *Pseudomonas aeruginosa* in Cystic Fibrosis (TORPEDO-CF): A Randomised Controlled Trial. Lancet Respir. Med. 8 (10), 975–986. 10.1016/S2213-2600(20)30331-3 33007285 PMC7606906

[B31] Imperi F. Tiburzi F. Visca P. (2009). Molecular Basis of Pyoverdine Siderophore Recycling in *Pseudomonas aeruginosa* . Proc. Natl. Acad. Sci. U. S. A. 106 (48), 20440–20445. 10.1073/pnas.0908760106 19906986 PMC2787144

[B32] Kelly N. M. Kluftinger J. L. Pasloske B. L. Paranchych W. Hancock R. E. W. (1989). *Pseudomonas aeruginosa* Pili as Ligands for Nonopsonic Phagocytosis by Fibronectin-Stimulated Macrophages. Infect. Immun. 57 (12), 3841–3845. 10.1128/IAI.57.12.3841-3845.1989 2572562 PMC259914

[B33] Kollaran A. M. Joge S. Kotian H. S. Badal D. Prakash D. Mishra A.. (2019). Context-Specific Requirement of Forty-Four Two-Component Loci in *Pseudomonas aeruginosa* Swarming. iScience 13, 305–317. 10.1016/j.isci.2019.02.028 30877999 PMC6423354

[B34] Koskenkorva-Frank T. S. Kallio P. T. (2009). Induction of *Pseudomonas aeruginosa Fhp* and *fhpR* by Reactive Oxygen Species. Can. J. Microbiol. 55 (6), 657–663. 10.1139/w09-024 19767835

[B35] Kumar P. Nagarajan A. Uchil P. D. (2018). Analysis of Cell Viability by the Lactate Dehydrogenase Assay. Cold Spring Harb. Protoc. 6. 10.1101/pdb.prot095497 29858337

[B36] Lai A. Tremblay J. Déziel E. (2009). Swarming Motility: A Multicellular Behaviour Conferring Antimicrobial Resistance. Environ. Microbiol. 11 (1), 126–136. 10.1111/j.1462-2920.2008.01747.x 18793317

[B37] Landman D. Bratu S. Kochar S. Panwar M. Trehan M. Doymaz M.. (2007). Evolution of Antimicrobial Resistance Among *Pseudomonas aeruginosa*, *Acinetobacter baumannii* and *Klebsiella pneumoniae* in Brooklyn, NY. J. Antimicrob. Chemother. 60, 78–82. 10.1093/jac/dkm129 17490999

[B38] La Rosa R. Johansen H. K. Molin S. (2019). Adapting to the Airways: Metabolic Requirements of *Pseudomonas aeruginosa* During the Infection of Cystic Fibrosis Patients. Metabolites 9 (10):234. 10.3390/metabo9100234 PMC6835255 31623245

[B39] Li G. Lu C. D. (2016). Molecular Characterization of LhpR in Control of Hydroxyproline Catabolism and Transport in *Pseudomonas aeruginosa* PAO1. Microbiology (Read) 162 (7), 1232–1242. 10.1099/mic.0.000300 27145750

[B40] Line L. Alhede M. Kolpen M. Kühl M. Ciofu O. Bjarnsholt T.. (2014). Physiological Levels of Nitrate Support Anoxic Growth by Denitrification of *Pseudomonas aeruginosa* at Growth Rates Reported in Cystic Fibrosis Lungs and Sputum. Front. Microbiol. 5, 554. 10.3389/fmicb.2014.00554 25386171 PMC4208399

[B41] Lovewell R. R. Patankar Y. R. Berwin B. (2014). Mechanisms of Phagocytosis and Host Clearance of *Pseudomonas aeruginosa* . Am. J. Physiol. Lung Cell Mol. Physiol. 306 (7), L591–L603. 10.1152/ajplung.00335.2013 24464809 PMC4116407

[B42] Luque-Almagro V. M. Gates A. J. Moreno-Vivian C. Ferguson S. J. Richardson D. J. Roldan M. D. (2011). Bacterial Nitrate Assimilation: Gene Distribution and Regulation. Biochem. Soc. Trans. 39, 1838–1843. 10.1042/BST20110688 22103536

[B43] Meylan S. Porter C. B. M. Yang J. H. Belenky P. Gutierrez A. Lobritz M. A.. (2017). Carbon Sources Tune Antibiotic Susceptibility in *Pseudomonas aeruginosa* via Tricarboxylic Acid Cycle Control. Cell Chem. Biol. 24, 195–206. 10.1016/j.chembiol.2016.12.015 28111098 PMC5426816

[B44] Moradali M. F. Ghods S. Rehm B. H. A. (2017). *Pseudomonas aeruginosa* Lifestyle: A Paradigm for Adaptation, Survival and Persistence. Front. Cell. Infect. Microbiol. 7, 39. 10.3389/fcimb.2017.00039 28261568 PMC5310132

[B45] Naren N. Zhang X.-X. (2021). Role of a Local Transcription Factor in Governing Cellular Carbon/Nitrogen Homeostasis in *Pseudomonas fluorescens* . Nucleic Acids Res. 49 (6), 3204–3216. 10.1093/nar/gkab091 33675669 PMC8034625

[B46] O’Brien S. Williams D. Fothergill J. L. Paterson S. Winstanley C. Brockhurst M. A. (2017). High Virulence Sub-Populations in *Pseudomonas aeruginosa* Long-Term Cystic Fibrosis Airway Infections. BMC Microbiol. 17 (1), 30. 10.1186/s12866-017-0941-6 28158967 PMC5291983

[B47] Ohman D. E. Cryz S. J. Iglewski B. H. (1980). Isolation and Characterization of *Pseudomonas aeruginosa* PAO Mutant That Produces Altered Elastase. J. Bacteriol. 142, 836–842. 10.1128/jb.142.3.836-842.1980 6769912 PMC294108

[B48] Olivares E. Badel-Berchoux S. Provot C. Prévost G. Bernardi T. Jehl F. (2020). Clinical Impact of Antibiotics for the Treatment of *Pseudomonas aeruginosa* Biofilm Infections. Front. Microbiol. 10, 2894. 10.3389/fmicb.2019.02894 31998248 PMC6962142

[B49] Palmer K. L. Aye L. M. Whiteley M. (2007). Nutritional Cues Control *Pseudomonas aeruginosa* Behavior in Cystic Fibrosis Sputum 189, 22, 8079–8087. 10.1128/JB.01138-07 PMC2168676 17873029

[B50] Palmer K. L. Mashburn L. M. Singh P. K. Whiteley M. (2005). Cystic Fibrosis Sputum Supports Growth and Cues Key Aspects of *Pseudomonas aeruginosa* Physiology. J. Bacteriol. 187 (15), 5267–5277. 10.1128/JB.187.15.5267-5277.2005 16030221 PMC1196007

[B51] Panayidou S. Georgiades K. Christofi T. Tamana S. Promponas V. J. Apidianakis Y. (2020). *Pseudomonas aeruginosa* Core Metabolism Exerts a Widespread Growth-Independent Control on Virulence. Sci. Rep. 10, 9505. 10.1038/s41598-020-66194-4 32528034 PMC7289854

[B52] Pang Z. Raudonis R. Glick B. R. Lin T. Cheng Z. (2019). Antibiotic Resistance in *Pseudomonas aeruginosa*: Mechanisms and Alternative Therapeutic Strategies. Biotechnol. Adv. 37 (1), 177–192. 10.1016/j.biotechadv.2018.11.013 30500353

[B53] Perinbam K. Chacko J. V. Kannan A. Digman M. A. Siryaporn A. (2020). A Shift in Central Metabolism Accompanies Virulence Activation in *Pseudomonas aeruginosa* . mBio 11 (2), e02730–e02718. 10.1128/mBio.02730-18 32156820 PMC7064766

[B54] Pletzer D. Mansour S. C. Wuerth K. Rahanjam N. Hancock R. E. W. (2017). New Mouse Model for Chronic Infections by Gram-Negative Bacteria Enabling the Study of Anti-Infective Efficacy and Host-Microbe Interactions. mBio 8 (1), e00140-17. 10.1128/mBio.00140-17 28246361 PMC5347345

[B55] Rehman A. Patrick W. M. Lamont I. L. (2019). Mechanisms of Ciprofloxacin Resistance in *Pseudomonas aeruginosa*: New Approaches to an Old Problem. J. Med. Microbiol. 68, 1–10. 10.1099/jmm.0.000873 30605076

[B56] Strateva T. Mitov I. (2011). Contribution of an Arsenal of Virulence Factors to Pathogenesis of *Pseudomonas aeruginosa* . Ann. Microbiol. 61, 717–732. 10.1007/s13213-011-0273-y

[B57] Thöming J. G. Tomasch J. Preusse M. Koska M. Grahl N. Pohl S.. (2020). Parallel Evolutionary Paths to Produce More Than One *Pseudomonas aeruginosa* Biofilm Phenotype. NPJ Biofilms Microbiomes 6, 2. 10.1038/s41522-019-0113-6 31934344 PMC6954232

[B58] Tschudin-Sutter S. Fosse N. Frei R. Widmer A. F. (2018). Combination Therapy for Treatment of *Pseudomonas aeruginosa* Bloodstream Infections. PLoS One 13 (9), e0203295. 10.1371/journal.pone.0203295 30235247 PMC6147480

[B59] Winstanley C. O’Brien S. Brockhurst M. A. (2016). *Pseudomonas aeruginosa* Evolutionary Adaptation and Diversification in Cystic Fibrosis Chronic Lung Infections. Trends Microbiol. 24 (5), 327–337. 10.1016/j.tim.2016.01.008 26946977 PMC4854172

[B60] Yang H. Shan Z. Kim J. Wu W. Li W. Zeng L.. (2007). Regulatory Role of PopN and Its Interacting Partners in Type III Secretion of *Pseudomonas aeruginosa* . J. Bacteriol. 189 (7), 2599–2609. 10.1128/JB.01680-06 17237176 PMC1855783

[B61] Yeung A. T. Y. Janot L. Pena O. M. Neidig A. Kukavica-Ibrulj I. Hilchie A.. (2014). Requirement of the *Pseudomonas aeruginosa* CbrA Sensor Kinase for Full Virulence in a Murine Acute Lung Infection Model. Infect. Immun. 82 (3), 1256–1267. 10.1128/IAI.01527-13 24379284 PMC3957987

[B62] Yeung A. T. Y. Torfs E. C. W. Jamshidi F. Bains M. Wiegand I. Hancock R. E. W.. (2009). Swarming of *Pseudomonas aeruginosa* is Controlled by a Broad Spectrum of Transcriptional Regulators, Including MetR. J. Bacteriol. 191 (18), 5592–5602. 10.1128/JB.00157-09 19592586 PMC2737960

[B63] Zarzycki-Siek J. Norris M. H. Kang Y. Sun Z. Bluhm A. P. McMillan I. A.. (2013). Elucidating the *Pseudomonas aeruginosa* Fatty Acid Degradation Pathway: Identification of Additional Fatty acyl-CoA Synthetase Homologues. PLoS One 8 (5), e64554. 10.1361/journal.pone.0064554 23737986 PMC3667196

