# Supplementary material for: NtrBC Selectively Regulates Host-Pathogen Interactions, Virulence, and Ciprofloxacin Susceptibility of Pseudomonas aeruginosa
Source: Front Cell Infect Microbiol. 2021 Jun 24;11:694789. doi: 10.3389/fcimb.2021.694789 (PMC8264665; doi:10.3389/fcimb.2021.694789)
Supplement: Supplementary file 1 [file DataSheet_1.docx]

NtrBC selectively regulates host-pathogen interactions, virulence and ciprofloxacin susceptibility of *Pseudomonas aeruginosa*

**Supplemental Information**

**
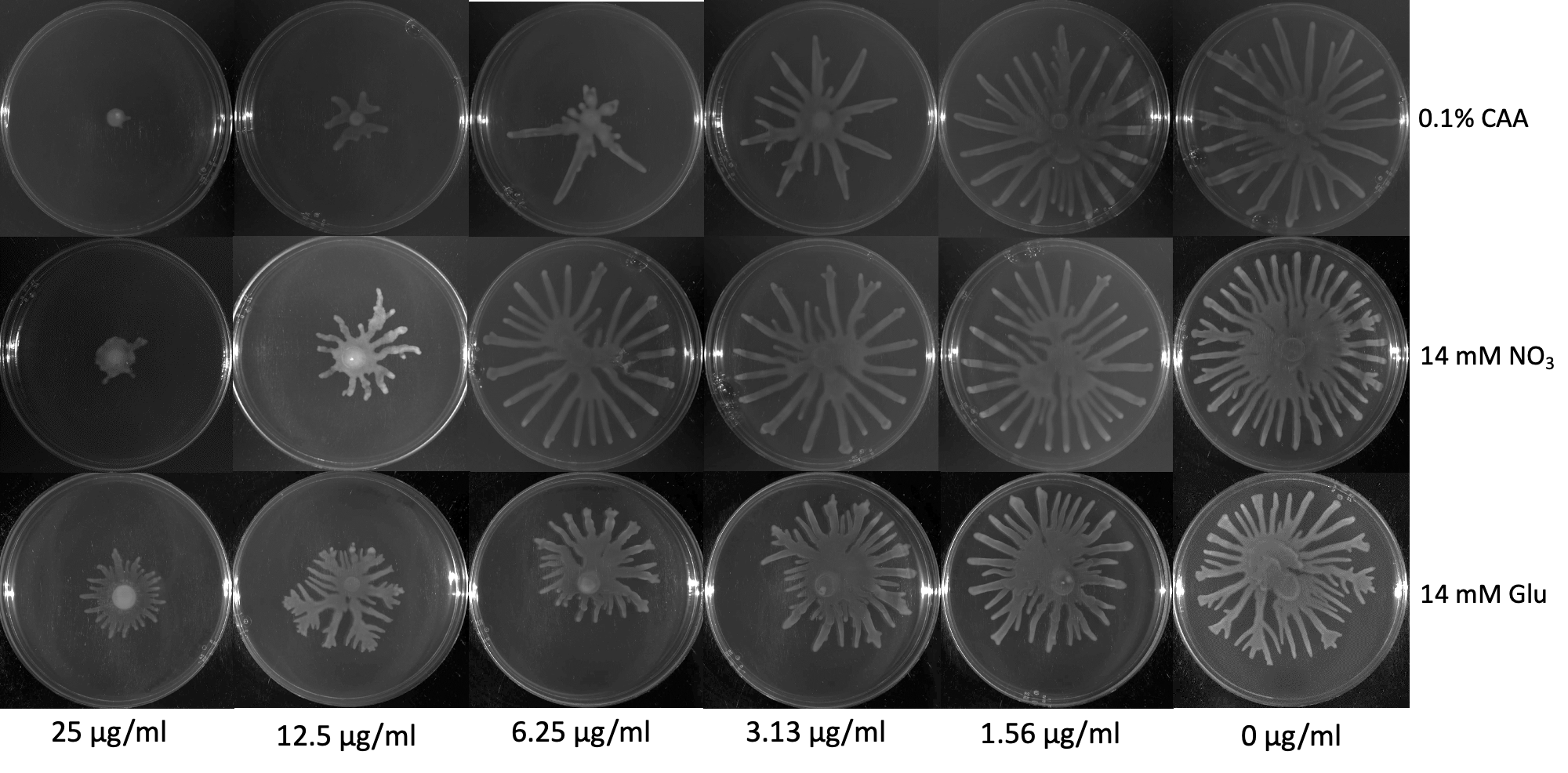
**

**Figure S1. *P. aeruginosa* PA14 swarming-mediated resistance to ciprofloxacin depends on nitrogen source.** Swarming assays were performed on BM2 supplemented with 0.4% glucose and 0.5% agar as well as casamino acids (CAA), nitrate (NO_3_^-^) or glutamate (Glu) in equimolar amounts. Ciprofloxacin was titrated (0-25 µg/ml) into the plate, as indicated on the abscissa. Swarming in the presence of ciprofloxacin was greatest when Glu was used as the nitrogen source, and weakest when CAA were used. Data are representative images of three independent experiments (*n* = 3) captured with a BioRad ChemiDoc.

**
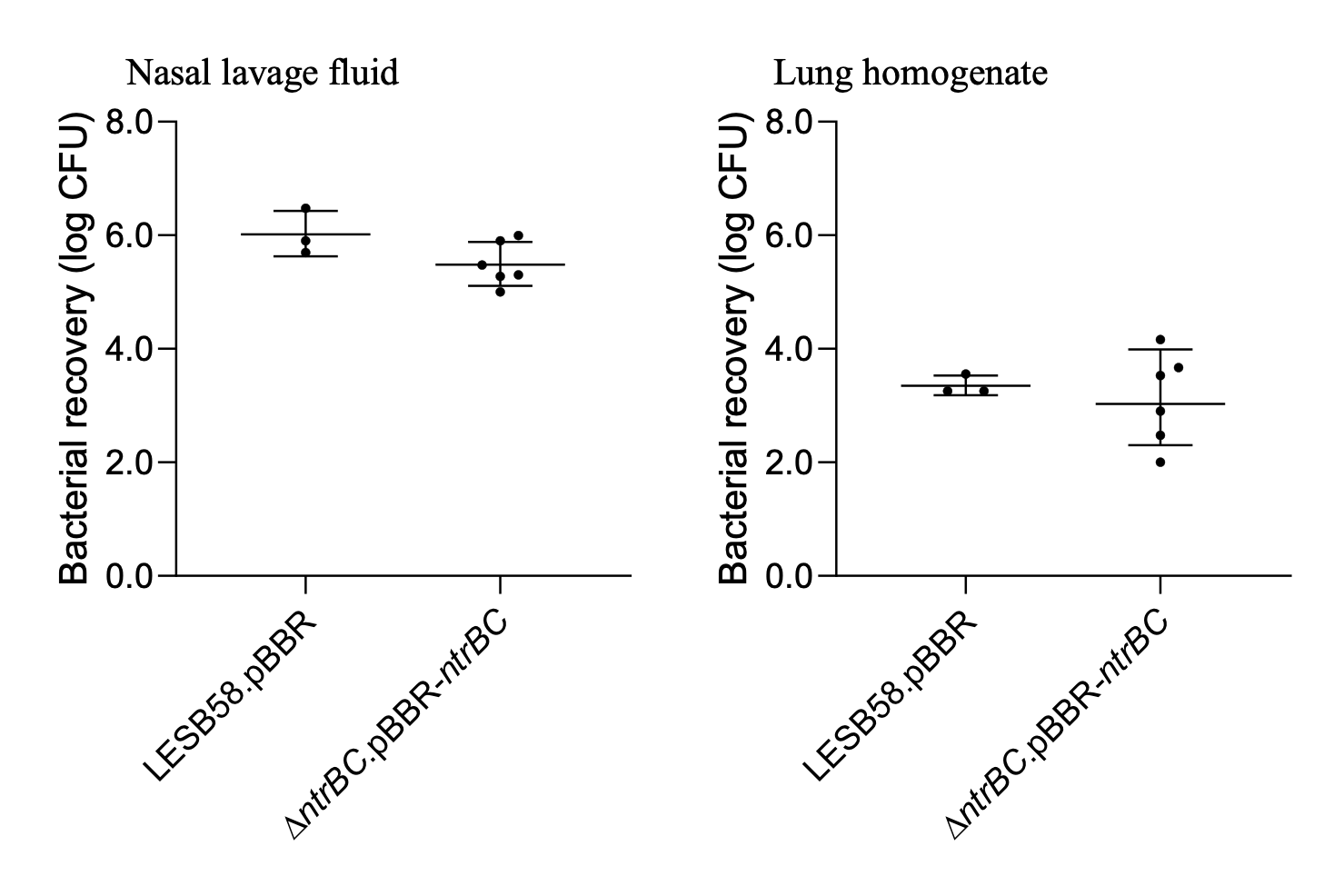
**

**Figure S2. Complementation of the nitrogen regulatory two-component system, NtrBC, restored infectivity of *P. aeruginosa* in a murine model of sinusitis*.*** Stationary-phase bacteria were inoculated dropwise in the left nare of C57Bl/6 mice (10^6^ CFU). 72 h later mice were euthanized, and lung tissue or nasal lavage fluid was collected for bacterial enumeration. Similar levels of bacteria were recovered from the lungs and nasal cavities of mice infected with *P. aeruginosa* with empty vector (pBBR1MCS-5) and vector containing *ntrBC*. Data are presented as geometric mean ± standard deviation for two independent experiments containing 1-3 biological replicates each (*n* = 3-6).

**Figure S3. Neutrophil mediated uptake of P. aeruginosa PA14 WT and ntrBC mutant was not significantly different.** Human neutrophils were isolated from whole blood using the StemCell EasySep kit (Vancouver, BC, Canada) and treated with mid-log phase bacteria at a multiplicity of infection (MOI) = 10. Cells were turned end over end and incubated at 37°C for 30 min. Cells were washed, treated with gentamicin (500 µg/ml) for 30 min, and washed again to remove any residual bacteria in the supernatant. Cell lysates at t = 0, 60 min were plated for bacterial enumeration following serial dilution. Data are presented as geometric mean ± standard deviation for three independent experiments containing 1 biological replicate each (*n* = 3). Mutants were taken up and cleared from neutrophils similarly to WT according to student’s t-test.

**Table S1. Bacterial strains and plasmids used in this study.** Gm = gentamicin.

| **Strain or plasmid** | **Relevant characteristics^a^** | **Ref. or Source** |
| --- | --- | --- |
| *Pseudomonas aeruginosa* | | |
| PA14 | *P. aeruginosa* UBCPP-PA14 WT | Rahme et al. 1995 |
| PA14 Δ*ntrB* | PA14 *ntrB* chromosomal deletion | Alford et al. 2020 |
| PA14 Δ*ntrC* | PA14 *ntrC* chromosomal deletion | Alford et al. 2020 |
| PA14 Δ*ntrBC* | PA14 *ntrBC* chromosomal deletion | Alford et al. 2020 |
| LESB58 | *P. aeruginosa* Liverpool Epidemic Strain B58 WT | Cheng et al. 1996 |
| LESB58 Δ*ntrB* | LESB58 *ntrB* chromosomal deletion | Alford et al. 2020 |
| LESB58 Δ*ntrC* | LESB58 *ntrC* chromosomal deletion | Alford et al. 2020 |
| LESB58 Δ*ntrBC* | LESB58 *ntrBC* chromosomal deletion | Alford et al. 2020 |
| Other motile species | | |
| NCIB 3610 | *Bacillus subtilis* | Bacillus Stock Center |
| O157:H7 | *Escherichia coli* | Nataro and Kaper, 1998 |
| ATCC 14028s | *Salmonella enterica* serovar Typhimurium | ATCC |
| Plasmids | | |
| pBBR1MCS-5 | Broad host-range cloning vector, Gm^r^ | Kovach et al. 1994 |
| pBBR.*ntrBC* | Cloned 2.51 kbp *ntrBC* gene, Gm^r^ | Alford et al. 2020 |

**Table S2.** **Swarming-mediated adaptive resistance to various antibiotics is conserved across species.** Bacteria (*B. subtilis* NCIB 3610, *E. coli* O157:H7, *P. aeruginosa* PA14 and *S. enterica* ATCC 14028s) harvested from batch culture (planktonic), swimming plates or swarming plates were adjusted to an OD = 0.01 and seeded in 96-well polypropylene plates containing MHB and antibiotic. 18-20 h later, wells were visually examined for turbidity indicating bacterial growth. Minimal inhibitory concentration (MIC) of antibiotics is expressed as the lowest concentration that inhibited growth in at least two of three replicates from three independent experiments (*n* = 3). Antibiotics: ciprofloxacin (Cip), tobramycin (Tb), chloramphenicol (Cap) and tetracycline (Tc).

| Organism | MIC (μg/ml) | | | | | | | | | | | |  |
| --- | --- | --- | --- | --- | --- | --- | --- | --- | --- | --- | --- | --- | --- |
|  | **MHB** | | | | **BM2 swim plate** | | | | **BM2 swarm plate** | | | |  |
|  | Cip | Tb | Cap | Tc | Cip | Tb | Cap | Tc | Cip | Tb | Cap | Tc | |
| *B. subtilis* | 3.13 | 1.56 | 3.13 | 3.13 | 6.25 | 0.78 | 3.13 | 3.13 | 12.5 | 1.56 | 6.25 | 12.5 | |
| *E. coli* | 12.5 | 3.13 | 3.13 | 3.13 | 12.5 | 12.5 | 1.56 | 1.56 | >200 | >200 | 25 | 100 | |
| *P. aeruginosa* | 3.13 | 3.13 | 12.5 | 6.25 | 3.13 | 3.13 | 25 | 12.5 | >200 | 50 | 100 | 50 | |
| *S. enterica* | 6.25 | 12.5 | 0.78 | 0.78 | 6.25 | 6.25 | 3.13 | <0.39 | 6.25 | 50 | 50 | 6.25 | |

**Table S3. Genes involved in synthesis of virulence factors were downregulated, whereas genes involved in macrophage uptake were upregulated, in NtrBC mutants.** Fastq and count files for all samples are available on the NCBI Gene Expression Omnibus (GEO) under accession number GSE145591. Analysis revealed differential expression of xx genes belonging to the ciprofloxacin resistome that were significantly downregulated in NtrB and/or NtrC mutants relative to PA14 WT. Data are expressed as mean fold-change (FC) values from three biological replicates. Blank cells indicate no change in expression.

| **PA14 ID** | **Name** | **Annotation** | **FC Δ*ntrB*** | **FC Δ*ntrC*** |
| --- | --- | --- | --- | --- |
| Synthesis of siderophores and phenazines^1^ | | | | |
| PA14_18020 | PA3578 | PhzF family phenazine biosynthesis protein |  | -1.7 |
| PA14_29650 | *ppyR* | psl and pyoverdine operon regulator | -4.1 | -4.8 |
| PA14_33270 | *pvdG* | PvdG | -4.0 | -7.6 |
| PA14_33280 | *pvdL* | PvdL | -4.3 | -6.1 |
| PA14_33530 | *fpvF* | FpvF | -2.5 | -4.0 |
| PA14_33540 | *fpvE* | FpvE | -8.1 | -6.5 |
| PA14_33550 | *fpvD* | FpvD | -4.6 | -6.6 |
| PA14_33560 | *fpvC* | FpvC | -3.4 | -5.8 |
| PA14_33570 | *fpvK* | FpvK | -6.4 | -8.0 |
| PA14_33590 | *fpvH* | FpvH | -5.5 | -5.9 |
| PA14_33600 | *fpvG* | FpvG | -2.7 | -4.2 |
| PA14_33630 | *pvdJ* | PvdJ | -2.4 | -2.4 |
| PA14_33650 | *pvdD* | pyoverdine synthetase D | -2.1 | -2.0 |
| PA14_33680 | *fpvA* | ferripyoverdine receptor | -1.9 | -2.8 |
| PA14_33690 | *pvdE* | pyoverdine biosynthesis protein PvdE |  | -3.3 |
| PA14_33710 | *pvdO* | PvdO | -5.1 | -3.7 |
| PA14_33810 | *pvdA* | L-ornithine N5-oxygenase | -2.5 | -3.5 |
| PA14_33820 | *pvdQ* | 3-oxo-C12-homoserine lactone acylase PvdQ | -4.1 | -5.9 |
| PA14_39980 | *qscR* | quorum-sensing control repressor | -3.4 | -3.4 |
| Macrophage uptake^2^ | | | | |
| PA14_09260 | *pchR* | transcriptional regulator PchR | 1.7 | 1.7 |
| PA14_09440 | *phzE2* | phenazine biosynthesis protein PhzE | 1.9 | 1.7 |
| PA14_09440 | *phzE1* | phenazine biosynthesis protein PhzE | 1.9 | 1.7 |
| PA14_14680 | PA3818 | extragenic suppressor protein SuhB | 1.5 | 1.5 |
| PA14_14820 | *ndk* | nucleoside diphosphate kinase | 1.6 |  |
| PA14_15970 | *rpsP* | 30S ribosomal protein S16 | 1.7 |  |
| PA14_20970 | PA3331 | cytochrome P450 | 1.7 | 1.5 |
| PA14_21000 | PA3329 | hypothetical protein | 1.7 |  |
| PA14_42250 | *pscL* | type III export protein PscL |  | 3.5 |
| PA14_42260 | *pscK* | type III export protein PscK |  | 4.2 |
| PA14_42270 | *pscJ* | type III export protein PscJ | 2.5 | 4.4 |
| PA14_42280 | *pscI* | type III export protein PscI | 3.0 | 4.2 |
| PA14_42290 | *pscH* | type III export protein PscH | 3.2 | 4.8 |
| PA14_42340 | *pscD* | type III export protein PscD |  | 3.5 |
| PA14_42350 | *pscC* | Type III secretion outer membrane protein PscC precursor | 3.0 | 4.9 |
| PA14_42360 | *pscB* | type III export apparatus protein |  | 5.5 |
| PA14_42380 | *exsD* | ExsD | 2.5 | 4.0 |
| PA14_42430 | *exsC* | ExsC, exoenzyme S synthesis protein C precursor. |  | 3.0 |
| PA14_42440 | *popD* | Translocator outer membrane protein PopD precursor | 7.2 | 11.7 |
| PA14_42450 | *popB* | translocator protein PopB | 7.5 | 12.7 |
| PA14_42460 | *pcrH* | regulatory protein PcrH | 7.0 | 9.4 |
| PA14_42470 | *pcrV* | type III secretion protein PcrV | 4.5 | 7.7 |
| PA14_42480 | *pcrG* | regulator in type III secretion | 4.6 | 8.7 |
| PA14_42500 | *pcrD* | type III secretory apparatus protein PcrD | 2.7 | 3.8 |
| PA14_42520 | *pcr3* | Pcr3 | 6.2 | 11.7 |
| PA14_42550 | *popN* | Type III secretion outer membrane protein PopN precursor | 5.4 | 9.1 |
| PA14_42570 | PA1697 | ATP synthase in type III secretion system | 4.5 | 7.9 |
| PA14_42660 | *pscU* | translocation protein in type III secretion | 3.4 | 6.2 |
| PA14_52230 | *pirA* | ferric enterobactin receptor PirA |  | 1.9 |
| PA14_53250 | *cbpD* | chitin-binding protein CbpD precursor | 2.3 | 1.9 |
| PA14_53250 | PA0852 | Uncharacterized protein | 2.3 | 1.9 |
| PA14_58600 | PA4516 | hypothetical protein |  | 1.7 |
| PA14_60400 | *rpsT* | 30S ribosomal protein S20 | 1.7 |  |
| PA14_61770 | *prs* | ribose-phosphate pyrophosphokinase |  | 1.6 |
| PA14_62350 | *phuR* | Heme/Hemoglobin uptake outer membrane receptor PhuR precursor | 1.6 | 2.2 |
| PA14_62780 | PA4746 | conserved hypothetical protein |  | 1.6 |
| PA14_72960 | PA5530 | C5-dicarboxylate transporter | 13.3 | 12.4 |

^1^Ref: Winsor et al. 2016. ^2^Ref: Felgner et al. 2020

**Supplemental References**

Alford MA, Baghela A, Yeung ATY, Pletzer D, Hancock REW. (2020). NtrBC regulates invasiveness and virulence of Pseudomonas aeruginosa during high-density infection. Front. Micro. 11:773. doi: 10.3389/fmicb.2020.00773.

Felgner S, Preusse M, Beutling U, Stahnke S, Pawar V, Rohde M, Brönstrup M, Stradal T, Häussler S. (2020). Host-induced spermidine production in motile *Pseudomonas aeruginosa* triggers phagocytic uptake. eLife. 9: e55744. doi: 10.7554/eLife.55744

Kovach ME, Elzer PH, Hill DS, Robertson GT, Farris MA, Roop RM 2nd, et al. (1995). Four new derivatives of the broad-host-range cloning vector pBBR1MCS, carrying different antibiotic-resistance cassettes. Gene. 166(1): 175–6.

Nataro JP, Kaper JB. (1998). Diarrheagenic *Escherichia coli*. Clin. Microbiol. Rev. 11(1): 142-201.

Winsor GL, Griffiths EJ, Lo R, Dhillon BK, Shay JA, Brinkman FS. (2016). Enhanced annotations and features for comparing thousands of *Pseudomonas* genomes in the *Pseudomonas* genome database. Nucleic Acids Res. 44(D1): D646-653. doi: 10.1093/nar/gkv1227
